# Use of dual genomic sequencing to screen mitochondrial diseases in pediatrics: a retrospective analysis

**DOI:** 10.1038/s41598-023-31134-5

**Published:** 2023-03-14

**Authors:** Teng-Hui Wu, Jing Peng, Li Yang, Yan-Hui Chen, Xiu-Lan Lu, Jiao-Tian Huang, Jie-Yu You, Wen-Xian Ou-Yang, Yue-Yu Sun, Yi-Nan Xue, Xiao Mao, Hui-Ming Yan, Rong-Na Ren, Jing Xie, Zhi-Heng Chen, Victor-Wei Zhang, Gui-Zhen Lyu, Fang He

**Affiliations:** 1 Department of Pediatrics, Xiangya Hospital Central South University, 87 Xiangya Road, Changsha, 410008 Hunan China; 2 Department of Pediatrics, Fujian Medical University Union Hospital, 29 Xinquan Road, Fuzhou, Fujian China; 3 Department of Pediatric Intensive Care Unit, Hunan Children’s Hospital, 86 Ziyuan Road, Changsha, Hunan China; 4 Department of Gastroenterology and Nutrition, Hunan Children’s Hospital, 86 Ziyuan Road, Changsha, Hunan China; 5 Department of Hepatopathy, Hunan Children’s Hospital, 86 Ziyuan Road, Changsha, Hunan China; 6 Department of Pediatric Intensive Care Unit, Guangdong Provincial People’s Hospital, Guangdong Academy of Medical Sciences (GAMS), 106 Zhongshan 2nd Road, Guangzhou, Guangdong China; 7 Department of Pediatrics, Brain Hospital of Hunan Province, 427 Furong Road, Changsha, Hunan China; 8 Department of Medical Genetics, Maternal,, Child Health Hospital of Hunan Province, 53 Xiangchun Road, Changsha, Hunan China; 9 Department of Pediatrics, The 900Th Hospital of Joint Logistic Support Force, PLA, Fuzhou, Fujian China; 10 Department of Pediatrics, The First Hospital of Hunan University of Chinese Medicine, 95 Shaoshan Road, Changsha, Hunan China; 11 Department of Pediatrics, The Third Xiangya Hospital, Central South University, 138 Tongzipo Road, Changsha, Hunan China; 12 Amcare Genomics Laboratory, Guangzhou, Guangdong China; 13 Department of Human and Molecular Genetics, Baylor College of Medicine, Houston, TX USA

**Keywords:** Neurological disorders, Medical genetics

## Abstract

Mitochondrial diseases (MDs) were a large group multisystem disorders, attributable in part to the dual genomic control. The advent of massively sequencing has improved diagnostic rates and speed, and was increasingly being used as a first-line diagnostic test. Paediatric patients (aged < 18 years) who underwent dual genomic sequencing were enrolled in this retrospective multicentre study. We evaluated the mitochondrial disease criteria (MDC) and molecular diagnostic yield of dual genomic sequencing. Causative variants were identified in 177 out of 503 (35.2%) patients using dual genomic sequencing. Forty-six patients (9.1%) had mitochondria-related variants, including 25 patients with nuclear DNA (nDNA) variants, 15 with mitochondrial DNA (mtDNA) variants, and six with dual genomic variants (*MT-ND6* and *POLG*; *MT-ND5* and *RARS2*; *MT*-*TL1* and *NARS2*; *MT-CO2* and *NDUFS1; MT-CYB* and *SMARCA2;* and *CHRNA4* and *MT-CO3*). Based on the MDC, 15.2% of the patients with mitochondria-related variants were classified as “unlikely to have mitochondrial disorder”. Moreover, 4.5% of the patients with non-mitochondria-related variants and 1.43% with negative genetic tests, were classified as “probably having mitochondrial disorder”. Dual genomic sequencing in suspected MDs provided a more comprehensive and accurate diagnosis for pediatric patients, especially for patients with dual genomic variants.

## Introduction

Mitochondrial Diseases (MDs) are a group of complex inborn metabolic defects caused by mutations in nuclear DNA (nDNA) or mitochondrial DNA (mtDNA), with a prevalence of approximately 1 in 5,000 and a carrier rate of approximately 1 in 200 individuals^1,2^. In general, mitochondrial disorders in paediatric patients are mainly caused by nuclear-encoded genes with severe manifestations^3^. The majority of protein subunits and proteins that maintain mitochondrial structure and function are encoded by approximately 1500 nuclear genes^4^. Moreover, the expression of mtDNA genes is regulated by nDNA. nDNA variants could exacerbate mtDNA variants, resulting in multiple deletions in mtDNA, which are mostly seen in adults^5,6^. In addition, even typical phenotypes, such as Leigh syndrome (LS), identifiable by neuroimaging, could be caused by numerous nDNA or mtDNA variants^7^. Overall, the relationship between nDNA and mtDNA is extremely complicated and nDNA and mtDNA variants are difficult to be distinguished based on clinical data. Due to the clinically and genetically heterogeneous features, selection of genomic tests and interpretation of variants are still challenging.

Respiratory chain enzyme activity assays in muscle biopsy or skin fibroblasts have been widely used for MDs diagnosis. However, biopsy as an invasive method is less acceptable to parents of paediatric patients^8,9^. Therefore, the diagnosis process of “genotype first” has become popular. Forny et al. recommended histological testing in cases of critical illness to confirm the pathogenicity of variants of unknown significance and clinical MDs, wherein molecular diagnosis was negative, and remained highly suspicious^10^. With the development of high-throughput sequencing technologies and decreasing costs, whole-exome sequencing and mtDNA sequencing are convenient for molecular diagnosis of MDs. A non-invasive, bigenomic sequencing approach comprising whole exome and mtDNA sequencing has been recommended as the first step in identifying MDs^11^.

In this study, we retrospectively evaluated the MD criteria (MDC)^12^ and molecular diagnostic yield of dual genomic sequencing in 503 unrelated paediatric patients from 9 hospitals, aiming to summarize the efficiency of dual genomic sequencing and propose future directions for diagnostic approaches.

## Materials and methods

### Patient cohort

A total of 503 paediatric patients undergoing dual genomic analysis were enrolled from nine hospitals belonging to the Mitochondrial Consortium Unit between January 2017 and March 2021. These patients had an unexplained neuromuscular system or multisystem progressive disorder, suspected to be a mitochondrial disorder. Clinical data of the patients, including phenotypic information, neuroimaging, routine biochemical test results, and MDC scores, were retrospectively analysed. We assessed their molecular diagnosis based on whether the variants explained the clinical features and clinical matching between our patients and previously reported ones, combining the American College of Medical Genetics (ACMG) classification of variants and segregation pattern. Genes were classified as mitochondria-related if they were associated with MDs documented in the literature^13^ or non-mitochondria-related when this was not the case.

### mtDNA sequencing

Peripheral blood samples were collected, and 3–5 μg of DNA was extracted using the salt extraction method with a DNA extraction kit, according to the manufacturer’s instructions (Mike Bio, China). Mitochondrial amplification was performed using mitochondria-specific primers^14^. Thereafter, the ultrasonic method was used to disrupt mitochondrial amplification products. Mitochondrial libraries were established using the KAPA HTP Library Preparation Kit (Kapa Biosystems Inc., Woburn, MA, USA). High-throughput sequencing was performed using a NextSeq 500 sequencer (Illumina, San Diego, CA, USA). The average sequencing depth was not less than 4000 × (Supplementary Table S1). After collecting data, bioinformatic analysis of the gene sequence was performed to confirm loci of pathogenic genes. The mtDNA variants were classified according to the mRNA^15^ and tRNA^16^ variant classification criteria.

### nDNA sequencing

Genomic DNA extracted from whole blood of the patients and their parents was sequenced using Illumina HiSeq X sequencers or Illumina Novaseq platforms with at least Q20 base quality and > 30 × mean nuclear coverage (Supplementary Table S1Sequences were compared to the human reference genome using the NextGENe software (SoftGenetics, State College, Pa, USA) for true and false variant identification. Single-nucleotide variants were retained and annotated by filtering high-frequency variants using population frequency (dbSNP, ExAC, and gnomAD) and literature databases (OMIM, HGMD, ClinVar, and MasterMind). Multiple software (SIFT, Polyphen2, MutationTaster, and AlamutVisual) were used to predict pathogenicity based on amino acid conservation, evolutionary predictions, and splicing site effects. Variants were confirmed by Sanger sequencing and classified according to the ACMG and Genomics guidelines^17^.

### CNV-sequencing

The experimental and analytical methods were performed as previously described^18^. Genomic DNA was extracted, followed by random fragmentation and short-read sequencing using an Illumina NextSeq500 or NovaSeq6000 sequencer (Illumina, San Diego, CA, USA). The resolution was 25 kb to 100 kb. Sequencing reads were cleaned by removing the reads when the base quality was less than Q20 and were mapped to the reference human genome version GRCh38/hg38. The high-variation regions, indicating highly homologous or repeated regions across different samples, were excluded from further analysis. The interpretation of copy number variations (CNVs) was based on the ACMG and ClinGen^19^.

### Data analysis and statistics

Statistical analysis was performed using GraphPad Prism 8.0 software (San Diego, CA, USA). Statistically significant differences between groups were analyzed using one-way ANOVA. Statistical significance was defined as *P < 0.05, **P < 0.005, ***P < 0.001, and ****P < 0.0001.

### Ethics approval and consent to participate//////

Ethical approval for this study was obtained from Xiangya Hospital Ethics Committee. Written informed consent was obtained from participants and parents or legal guardians of any participant under the age of 16. All methods were performed in accordance with the relevant guidelines and regulations.

### Consent for publication

This study does not include any personal information leading to the identification of any participants.

## Results

### Dual genome test results

**Figure 1:**
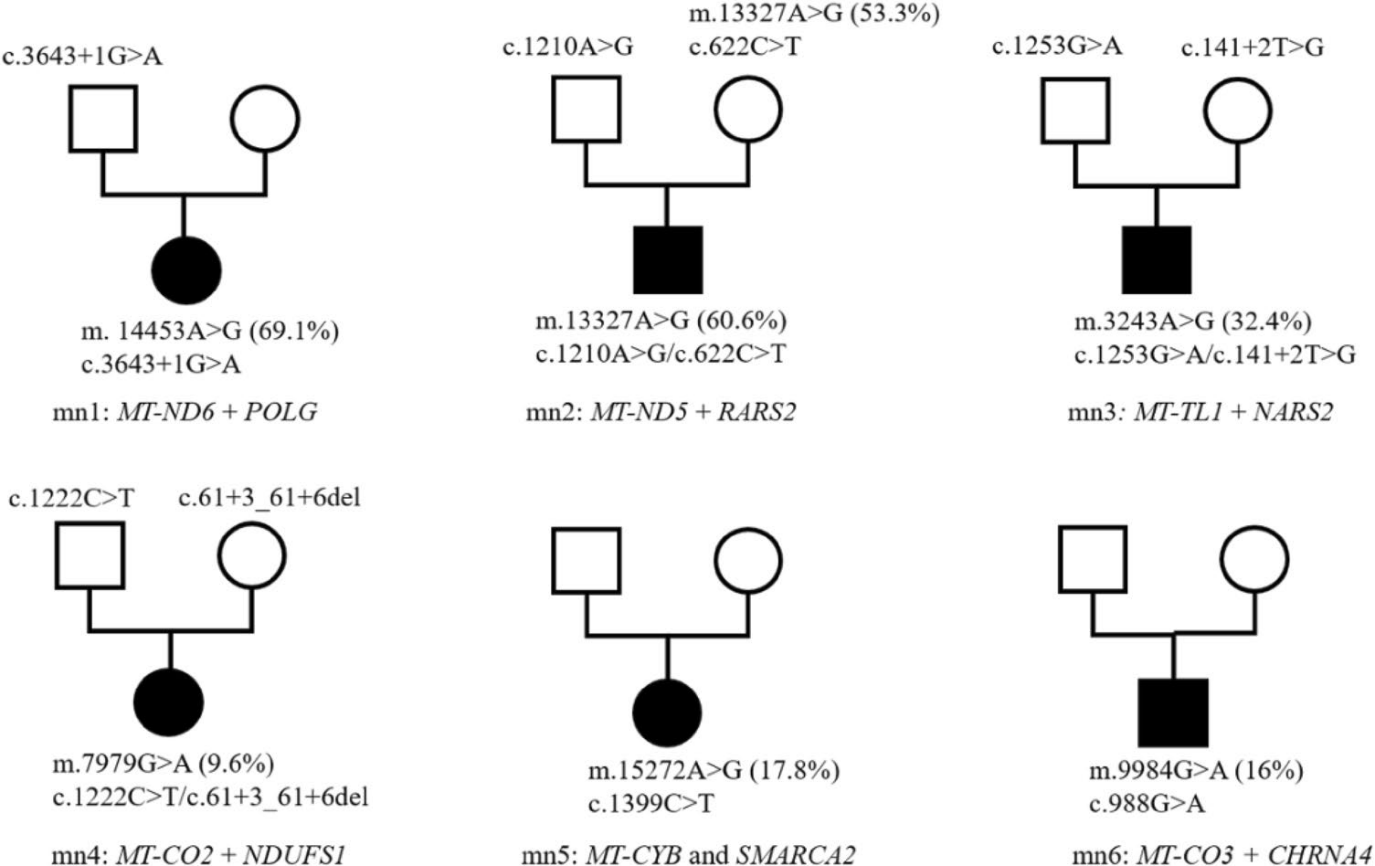
Family pedigree of six patients with dual genomic variants.

Among the 503 patients, mitochondria-related variants were identified in 46 (8.5%) (Table 1). In addition, 25 patients presented with variants in nDNA-encoded MDs genes, 15 with mtDNA variants, and six harboured both mitochondria-related nDNA and mtDNA variants. The nuclear-encoded MDs genes included *POLG* (n = 2)*, GTPBP3* (n = 2)*, ETFDH3* (n = 2)*, HIBCH* (n = 2), *AARS2, ACAD9, AIFM1, CARS2, COQ4, DNM1L, GFM1, HSD17B10, LIPT1, NAXE, OPA3, NDUFAF5, PANK2, PDHB, TWNK, WARS2,* and *PDHA1*. Furthermore, mtDNA genes included *MT-TL1*(n = 7)*, MT-ATP6*(n = 4)*, MT-ND1, MT-ND3, MT-ND4, MT-CO2* and a deletion m.10947-15362del*.* Four patients harboured dual genomic variants related to the mitochondria (Fig. 1), *MT-ND6* and *POLG*; *MT-ND5* and *RARS2*; *MT*-*TL1* and *NARS2*; and *MT-CO2* and *NDUFS1*. Two patients presented with mtDNA and non-mitochondria-related nDNA variants (Fig. 1): *MT-CYB* and *MARCA2* and *CHRNA4* and *MT-CO3*.

**Table 1:** Summary of clinical features of patients with mtDNA or/and nDNA variants.

Patient	Gender	Gene	Inheritance pattern	Variant	Zygosity	Origin	Age of onset (years)	Lactate (mmol/L)	MRI	Clinical features	ACMG	Evidence	Final clinical diagnosis	Novel mutation
**nDNA**														
n6	F	*HIBCH*	AR	c.1118A > G (p.N373S); c.810-4A > G	com.het	parental	10.2	8.57	Normal	Psychological and behavioral disorder	VUS/VUS	PM2 + PP3/PM2 + PP3	Behavioral abnormality	Y/Y
n10	M	*PANK2*	AR	c.1355A > G (p.D452G)	hom	parental	1	3.38	Basal ganglia involvement	DD, ataxia, retinitis pigmentosa	VUS	PS1 + PP4	Pantothenate kinase-associated neurodegeneration	N
n14	F	*AARS2*	AR	c.2682 + 5G > A; c.331G > C (p.A111P)	com.het	parental	1	2.9	Normal	Seizure, myoclonus, developmental delay, ataxia	VUS/VUS	PM2 + PP3/PM2 + PP3	Epileptic encephalopathy	N/N
n22	F	*GFM1*	AR	c.2167 T > C (p.C723R); c.539delG (p.G180Afs*11)	com.het	parental	7	5.83	Basal ganglia involvement	Muscle weakness, dystonia	VUS/P	PM2 + PM3 + PP4/PVS1 + PS1 + PM2	Leigh syndrome	Y/N
n26	M	*AIFM1*	XLR	c.1084A > C (p.K362Q)	hem	maternal	at birth	9.39	Thin corpus callosum	DD, hypertonia, microcephaly	VUS	PM2 + PP2	GDD	Y
n33	F	*HSD17B10*	XLD	c.628C > G (p.P210A)	het	de novo	at birth	10.22	Normal	DD	LP	PS2 + PM2 + PP3	HSD10 mitochondrial disease	Y
n20	M	*CARS2*	AR	c.323 T > G (p.F108C); c.1036C > T (p.R346W)	com.het	parental	2	2.74	Cortical atrophy	Seizure, psychological and behavioral disorder	VUS/VUS	PM2 + PP3/PM2 + PP3	Epileptic encephalopathy	Y/Y
n54	M	*HIBCH*	AR	c.958A > G (p.K320E); c.439-2A > G	com.het	parental	1.5	6.3	Basal ganglia involvement	Developmental regression, myoclonus, feeding difficulties	VUS/LP	PM2_P + PP3/ PVS1_M + PM3 + PP3	3-hydroxyisobutryl-CoA hydrolase deficiency	Y/Y
n59	F	*POLG*	AR	c.2890C > T (p.R964C); c.2584G > A (p.A862T)	com.het	parental	13	2.74	Cortical abnormalities	Seizure, tremer, liver disorder	VUS/VUS	PM3 + PM2_P + PP3/ PM3 + PM2_P + PP3	Alpers syndrome	N/N
n62	M	*COQ4*	AR	c.550 T > C (p.W184R); c.743 T > C (p.L248P)	com.het	parental	2	3.31	Basal ganglia involvement	DD, hypertonia, feeding difficulties	VUS/VUS	PM3 + PM2_P_PP3/PM2_P + PP3	Primary coenzyme Q10 deficiency	Y/Y
n89	F	*OPA3*	AR	c.123C > G (p.I41M)	hom	parental	1	1.23	Cerebellar atrophy	DD, seizure, ataxia	VUS	PM2_P + PP3	DD, seizure, ataxia	N
n98	F	*GTPBP3*	AR	c.187C > T (p.R63*); c.776A > G (p.N259S)	com.het	parental	at birth	6.94	Bilateral thalamus and left cortical involvement	DD, seizure	P/VUS	PVS1 + PM2.PP3/PM2 + PM3 + PP3	Combined oxidative phosphorylation deficiency 23	N/N
n108	F	*PDHA1*	XLD	c.901C > G(p.R301G)	het	de novo	0.5	3	Basal ganglia involvement	Muscle weakness	P	PS3 + PM2_P + PP3 + PP1_S	Pyruvate dehydrogenase E1-alpha deficiency	Y
n111	M	*WARS2*	AR	c.751 T > C (p.F251L)	hom	parental	1	4.68	Na	DD, seizure, tic disorder, ataxia, liver disorder	VUS	PM2_P + PP3	NEMMLAS	Y
n115	M	*LIPT1*	AR	c.302G > A (p.S101N); c.316G > A (p.V106I)	com.het	parental	4.6	1.5	Involvement of brainstem and thalamus	DD, gastrointestinal disorder	VUS/VUS	PM2_P + PP3/ PM2_P + PP3	Lipoyltransferase 1 deficiency	Y
n116	M	*ACAD9*	AR	c.1693-1G > A; c.1237G > A (p.E413K)	com.het	parental	0.3	1.9	Normal	Cardiovascular disorder, respiratory distress	VUS/LP	PVS1_M + PM2_P/PS3 + PM3 + PM2_P + PP3	Mitochondrial complex I deficiency	Y
n126	F	*PDHB*	AR	c.97-3C > G	hom	parental	2	8.67	White matter abnormalities	Ptosis, DD, seizure	VUS	PM2_P + PP3	Pyruvate dehydrogenase E1-beta deficiency	Y
n129	F	*DNM1L*	AD	c.1207C > T (p.R403C)	het	de novo	4.4	3.3	Involvement of brainstem and thalamus	Seizure, seizure status, coma	P	PS3 + PS2_M + PM2_P + PP1_M + PP3	Encephalopathy, lethal, due to defective mitochondrial peroxisomal fission 1	N
n130	M	*NAXE*	AR	c.473G > C (p.C158S); c.490C > A (p.P164T)	com.het	parental	2	Na	White matter abnormalities	DD, seizure	VUS/VUS	PM2_P + PP3/ PM2_P + PP3	Encephalopathy, progressive, early-onset, with brain edema and/or leukoencephalopathy	Y
n155	M	*POLG*	AR	c.2890C > T (p.R964C); c.2584G > A (p.A862T)	com.het	parental	5	Na	Cortical abnormalities	Seizure, muscle weakness, headache	VUS/VUS	PM3 + PM2_P + PP3/ PM3 + PM2_P + PP3	Alpers syndrome	Y
n167	M	*GTPBP3*	AR	c.413C > T (p.A138V); c.509_510delAG (p.E170Gfs*42)	com. het	parental	at birth	26	Na	respiratory failure	VUS/LP	PM3 + PM2_P + PP3/ PVS1 + PM2_P	Combined oxidative phosphorylation deficiency 23	Y
n179	M	*ETFDH*	AR	c.886G > T (p.G296C); c.1773_1774delAT (p.C592*)	com.het	parental	0.5	2	Normal	Muscle weakness, gastrointestinal disorder, impaired vision	VUS/VUS	PM2_P + PP3/ PVS1_M + PM2_P	Multiple acyl-CoA dehydrogenase deficiency	N/ N
n180	M	*NDUFAF5*	AR	c.752 T > G (p.M251R); c.155A > C (p.K52T)	com.het	parental	0.6	4.61	White matter abnormalities	Developmental regression	LP/LP	PM3_S + PM2_P + PP3/ PM3_S + PM2_P + PP3	Cavitating Leukoencephalopathy	N
2n4	M	*ETFDH*	AR	c.250G > A (p.A84T); c.2 T > G (p.M1?)	com.het	parental	0.5	Na	Na	Muscle weakness, liver disorder, gastrointestinal disorder	LP/VUS	PS3 + PM2 + PP3 + PPM2 + PP3	Combined oxidative phosphorylation deficiency 23	N/N
2n5	M	*TWNK*	AD	c.1421G > C (p.W474S)	het	maternal	at birth	5	Na	Ptosis	LP	PM2_P + PP3 + PM5 + PM6	Congenital myasthenic syndrome	Y
		*CHRNB1*	AD	c.1394 T > C (p.M465T)	het	maternal					VUS	-		Y
**mtDNA**														
m1	M	*MT-TL1*	maternal	m.3243A > G (71.2%)	71.20%	maternal (23.6%)	14	Na	Occipital cortex involvement with calcification	Seizure, headache, vomiting	P	PM2 + PP3-B + PS5 + PS2 + PS3	MELAS	N
m3	F	*MT-TL1*	maternal	m.3243A > G	66.20%	maternal (24.0%)	6.3	6.47	Cortical and basal ganglia involvement	Seizure, headache, vomiting	P	PM2 + PP3-B + PS5 + PS2 + PS3	MELAS	N
m4	F	*MT-TL1*	maternal	m.3243A > G	71.70%	maternal (24.4%)	4.7	9.33	Cortical, basal ganglia and thalamus involvement	Seizure, behavioral disorder	P	PM2 + PP3-B + PS5 + PS2 + PS3	MELAS	N
m6	F	*MT-ND3*	maternal	m.10197G > A	99.60%	de novo	0.5	3.5	Basal ganglia, thalamus and brainstem involvement	Developmental regression, seizure	LP	PP3-B + PP4 + PM9 + PM8	Leigh syndrome	N
m8	M	*MT-TL1*	maternal	m.3243A > G	66.10%	maternal (24.4%)	1	6.5	Basal ganglia involvement	Seizure, ID, diabetes	P	PM2 + PP3-B + PS5 + PS2 + PS3	Leigh syndrome	N
m9	M	*MT-ATP6*	maternal	m.9176 T > C	99.50%	maternal (88.2%)	8	4.49	Basal ganglia and brainstem involvement	Ptosis, muscle weakness, dysuria, tachycardia, tachypnea	P	PP3-A1 + PP3-B + PS1 + PM5 + PM9 + PM10 + PP4	Leigh syndrome	N
m10	M	*MT-TL1*	maternal	m.3243A > G	72.40%	de novo	12.6	7.33	Cortical abnormalities	Seizure	P	PM2 + PP3-B + PS5 + PS2 + PS3	MELAS	N
m12	M	*MT-TL1*	maternal	m.3243A > G	74.20%	maternal (10.2%)	8.7	4.73	Cortical abnormalities	Headache, vomiting, impaired vision	P	PM2 + PP3-B + PS5 + PS2 + PS3	MELAS	N
m13	M	*MT-ND1*	maternal	m.3761C > A	81.40%	0%	0.6	5.31	Ventriculomegaly	West syndrome	LP	PM2 + PM9	West syndrome	N
m14	M	*mtDNA (tissue)*	maternal	m.10947-15362del	/	Na	11	3.68	Na	Ptosis, growth restriction, skeletal muscle biopsy showing ragged red fibers	P	PM2 + PVS1 + PP4	KSS	Y
m16	F	*MT-TL1*	maternal	m.3243A > G	66.40%	de novo	8	6.69	Basal ganglia involvement	Developmental delay	P	PM2 + PP3-B + PS5 + PS2 + PS3	Leigh syndrome	N
m17	M	*MT-CO2, MT-ATP6*	maternal	m.7929G > A; m.9035 T > C	13.9%, 99.5%	de novo, de novo	1	3.3	Basal ganglia and brainstem involvement	Muscle weakness, hearing loss	VUS/LP	PM2 + PM9/PM2 + PM9 + PP4	Leigh syndrome	Y
m19	M	*MT-ATP6*	maternal	m.9176 T > C	99.80%	maternal (99.2%)	7	2.07	Abnormal signal foci around the midbrain aqueduct	ptosis, dysuria	P	PP3-A1 + PP3-B + PS1 + PM5 + PM9 + PM10 + PP4	Leigh syndrome	N
mn6	M	*MT-ATP6*	maternal	m.8993 T > C	99.50%	maternal (10.0%)	1.7	3.3	Basal ganglia involvement	muscle weakness, easy fatigability	P	PM2 + PP3-B + PS3 + PM5 + PS2	Leigh syndrome	N
mn9	M	*MT-ND4*	maternal	m.11778G > A	99.40%	maternal (99.4%)	0.6	3	Normal	Vision loss	P	PP3-A1 + PP3-B + PS1 + PS3	LHON	N
**mtDNA + nDNA**														
mn1	F	*MT-ND6*	maternal	m.14453A > G	69.10%	de novo	4.9	6.2	Cortical and basal ganglia involvement	Seizure	P	PM2 + PP3-B + PS4_M + PS2	MD	N
		*POLG*	AD/AR	c.3643 + 1G > A	het	paternal					VUS	PM2_P + PVS1_M		Y
mn2	M	*MT-ND5*	maternal	m.13327A > G	60.60%	maternal (53.3%)	at birth	4.13	Ventriculomegaly	DD, seizure, microcephalus	VUS	BS1 + BS4	EIEE	Y
		*RARS2*	AR	c.1210A > G (p.M404V); c.622C > T (p.Q208*)	com. het	parental					VUS/LP	PM2_P + PP3-B2(+ PP4) / PM2_P + PVS1(+ PP4)		Y/Y
mn3	M	*MT-TL1*	maternal	m.3243A > G	32.40%	de novo	2	1.67	Normal	Developmental regression, seizure, hearing loss	P	PM2 + PP3-B + PS5 + PS2 + PS3	MD	N
		*NARS2*	AR	c.1253G > A (p.R418H); c.141 + 2 T > G	com. het	parental					VUS/LP	PM2_P + PP3/ PVS1 + PM2_P		N/Y
mn4	F	*MT-CO2*	maternal	m.7979G > A	9.60%	de novo	at birth	6.52	Basal ganglia and brainstem involvement, ventriculomegaly	Muscle weakness, growth restriction	VUS	PM2 + BP4	Leigh syndrome	Y
		*NDUFS1*	AR	c.1222C > T(p.R408C); c.61 + 3_61 + 6delGAGT	com.het	parental					VUS/VUS	PM3_S + PM2_P + PP3/ PM2_P + PP3		N/Y
mn5	M	*MT-CYB*	maternal	m.15272A > G	17.80%	de novo	1.9	6.81	Hippocampus involvement	Development regression, seizure, dysarthria	VUS	PP3-B	Epileptic encephalopathy	N
		*SMARCA2*	AD	c.1399C > T (p.R467W)	het	de novo					LP	PS2 + PM2_P + PP3		Y
mn6	F	*CHRNA4*	AD	c.988G > A (p.V330M)	het	paternal	2.1	1.31	Normal	seizure	VUS	PM2_P + PP3	Epilepsy	Y
		*MT-CO3*	maternal	m.9984G > A	16%	de novo					VUS	PP7 + PS6		N

F, female; M, male; Na, not available; hom. homozygote; het, heterozygote; com. het. compound heterozygote; hem, hemizygote; DD, development delay; ID, intellectual disorder; NEMMLAS, neurodevelopmental disorder, mitochondrial, with abnormal movements and lactic acidosis, with or without seizures; GDD, global developmental delay; MELAS, mitochondrial myopathy, encephalopathy, lactic acidosis, and stroke-like episodes; LHON, Leber's hereditary optic neuropathy; MD, mitochondrial disease; EIEE, early onset epileptic encephalopathy; KSS, Kearns-Sayre syndrome; ACMG, American College of Medical Genetics and Genomics; P, pathogenic; LP, likely pathogenic; VUS, variant of uncertain significance.

Family history: n111, father had seizure; 2n5, mother, mother's sister, father, two father's sisters, and grandmother have ptosis; m8, mother had diabetes.

131 patients (26.0%) were found to have non-mitochondria-related variants, including 13 patients with pathogenic CNVs (Supplementary Table S2 and S3). Dual genomic analysis failed to identify definitive causative variants in 326 patients (64.8%). A few patients harboured low level of heteroplasmic mtDNA variants in blood, such as m.4358G > A (8.60%), m.10437G > A (blood 3.00%, urine 0%), m.594insC (21.80%), m.15867A > G (9.20%), m.5703G > A (2.50%), and m.3250 T > C (6.60%). Some variants of uncertain significance, such as *PMP22* (c.235 T > C) in infantile spasms, *KCNH5* (c.976G > T) in epilepsy, and *SEMA3E*, *PLEC* in suspected MDs.

### Patient demographics and clinical presentations

There was a male preponderance in 299 boys (59.4%) and 204 girls (40.6%) were included. A male to female ratio of 2.75 was observed in patients with mtDNA variants (Table 2). The median age of onset was 6.3 years (range, 0.5–14 years) in patients with mtDNA variants and one year (range, 0–13 years) in those with mitochondria-related nDNA variants. The patients with non-mitochondria-related variants (median, 0.5 years) and negative genetic test (median, one year) had an even younger age at first presentation than did those with mtDNA defects. The first symptoms included seizures; developmental delay or regression; muscle weakness; failure to thrive; ptosis; vomiting; increased alanine transaminase/aspartate transaminase; ataxia; headache; and visual signs. Seizures and developmental delay were the two most common symptoms in mitochondrial and non-mitochondrial patients in our cohort.

**Table 2:** Summary of demographics and clinical presentations in this study.

Groups	Mitochondria-related			Non-mitochondria-related	Negative
	mtDNA	nDNA	mtDNA + nDNA		
**Gender**					
Male	11	15	3	66	204
Female	4	10	3	65	122
Median age of onset (years)	6.3	1.0	1.2	0.5	1.0
Serum lactate (Mean ± SD, mmol/L)	5.0 ± 2.0	5.7 ± 5.4	4.4 ± 2.5	3.3 ± 3.28	2.80 ± 2.02

The serum lactate level in patients with mtDNA, mitochondria-related nDNA, and dual genomic variants were 5.0 ± 2.0 mmol/L, 5.7 ± 5.4 mmol/L, and 4.4 ± 2.5 mmol/L, respectively. The patients with non-mitochondria-related variants and negative genetic tests showed a lower lactate level of 3.3 ± 3.28 mmol/L and 2.80 ± 2.02 mmol/L, respectively (Table 1). Compared with the patients with negative genetic result, those with mtDNA (*P* = 0.0076) or mitochondria-related nDNA (*P* = 0.0483) variants showed significantly higher lactate level. In addition, the patients with mitochondria-related nDNA variants had significantly higher lactate level than that with non-mitochondria-related nDNA variants (*P* = 0.0076).

Neuroimaging data were available in 386 patients, 177 of whom exhibited normal brain magnetic resonance imaging (MRI) findings. The most frequent neuroradiological presentations included white matter (14.0%), basal ganglia (9.2%), cortical abnormalities (6.1%), cerebral atrophy (5.6%), cerebellar atrophy (3.9%), corpus callosum (3.6%), and brainstem and thalamic (2.9%) involvements. Less common presentations include optic atrophy, ventriculomegaly, and cysts. Basal ganglia characteristics were observed in the most mitochondrial patients (32.6%).

### Mitochondrial disease criteria

**Figure 2:**
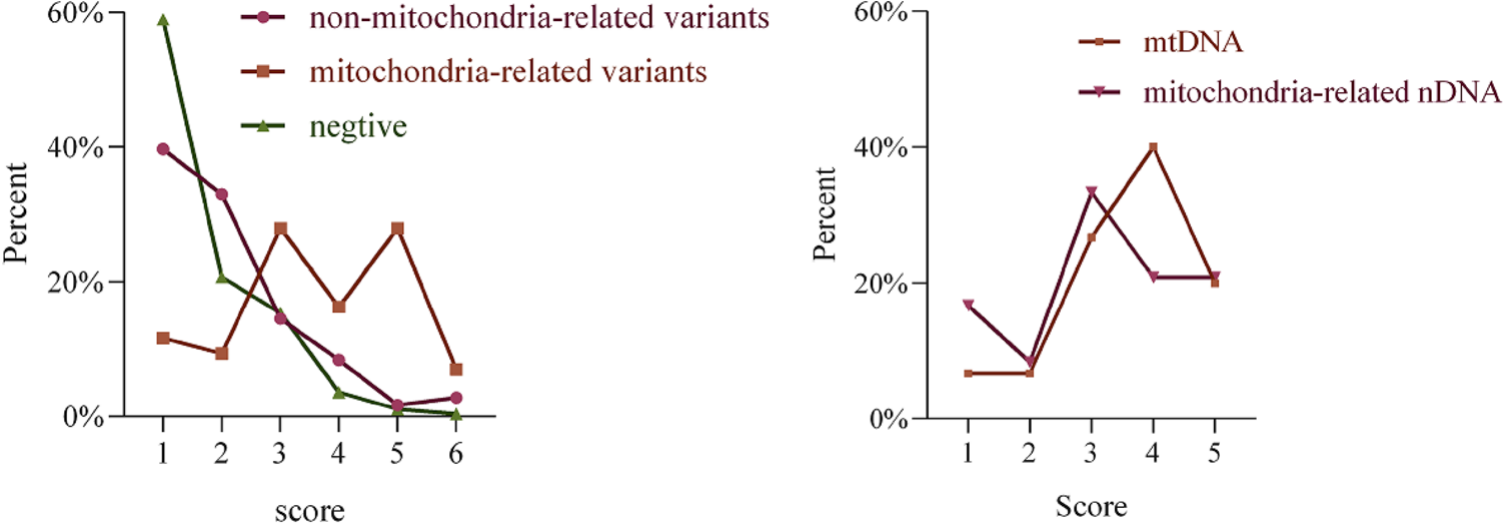
Percentages distribution of different groups referring to MDC scores. Left, comparison of three groups including negative, mitochondria-related and non-mitochondria-related nDNA variants. Right, comparison between mtDNA and mitochondria-related nDNA variants.

According to the MDC, 15.2%, 52.2% and 32.6% of the patients with mtDNA or mitochondria-related nDNA variants, were classified into “unlikely to have mitochondrial disorder” (score 1), “possibly having mitochondrial disorder” (score 2 to 4) and “probably having mitochondrial disorder” (score 5 to 7), respectively (Fig. 2). A comparison of the MDC scores between mtDNA and mitochondria-related nDNA variants is shown in Fig. 2. 42.6% and 18.7% of patients whose causative variants were not detected were determined as “unlikely to have mitochondrial disorder” and “possibly having mitochondrial disorder”. In patients with non-mitochondria-related variants, the corresponding percentages were 39.0% and 39.0%. A few confirmed mitochondrial cases had low MDC scores due to an organ-specific presentation, such as Leber hereditary optic neuropathy (LHON), progressive external ophthalmoplegia, cerebellar ataxia, or a single clinical symptom, such as epileptic encephalopathy without characteristic MRI or lactate level findings. The importance of the parts that might have been missed because of low scores should be noted.

In addition, there were a few noteworthy genes in the patients with high MDC scores. A few cases of non-mitochondria-related variants were evaluated as probably having mitochondrial disorders, such as variants in *MMACHC*, *MCEE*, *FOLR1* and *G6PC* genes, which were grouped as causative genes of metabolic diseases affecting multiple systems.

A few cases of high MDC scores in which confirmed causative variants could not be detected require further study. An eight-year-old girl presenting with developmental delay, vomiting, and abnormal behaviour whose examination showed hypoglycaemia, high serum lactate (5.04 mmol/L), bilateral basal ganglia involvement, and a de novo variant c.760G > C (p.E254Q) of *SEMA3E* gene was identified. A girl with a ventricular sepal defect presented with seizures after fever, and examination showed CK 5833U/L, CKMB 369.9U/L, LDH 1876U/L, and bilateral basal ganglia involvement on brain MRI. However, no mitochondria-related variant other than the *PLEC* gene with c.10947C > A (p.F3649L) and c.7603C > T (p.R2535C) was identified.

## Discussion

A two-step next-generation sequencing approach, by which patients were evaluated based on whole exome sequencing after excluding pathogenic variants in the mtDNA, was frequently used in previous cohort^20,28^. Simultaneous sequencing of the dual genome, including mtDNA genome-wide sequencing combined with whole-exon sequencing of trios, is not common in paediatric cohorts of MDs. Recently, whole-genome sequencing has been used to determine the gene-disease association of MDs^21,22^. Dual genomic sequencing was a choice before the widespread use of whole genome sequencing, which was helpful for comprehensive diagnosis and establishment of a mitochondrial disease knowledge base^23^.

MDs in children is mainly caused by nDNA variation, accounting for 75–95%^24,25^. In China, mtDNA variations accounted for the main part (63%) of MDs in children in a previous study^26^. In addition, similar proportion was found in our previous local cohort^27^. The explanation might be the selection bias of the detection method and limitation of targeted gene panel sequencing in previous years. Because conditional MDs such as LS, mitochondrial encephalomyopathy with lactate acidosis and stroke-like episodes (MELAS), and LHON are relatively easy to catch the attention of physicians, targeted gene panel or mtDNA hotspot tests are unlikely to detect novel genes or variants. In this study, dual genomic sequencing provided more accurate data that nDNA variants (25/46) accounted for a larger proportion than did mtDNA variants (15/46) in MDs.

Here, we describe the findings in 503 routine diagnostic nDNA sequencing combined with mtDNA genome-wide sequencing in patients with a suspected MD. Overall, a molecular diagnosis was established in 177 patients (34.5%) in this cohort, which was similar to a finding in a previous study with more stringent inclusion criteria^28^. Similarly, 31% of participants with suspected MDs under 18 years old received a molecular diagnosis by using whole genome sequencing^22^. In a previous study, 5.3%, 37.2%, and 16.8% of 113 patients with suspected MD were identified with mtDNA, mitochondria-related nDNA, and non-mitochondria-related nDNA variants, respectively^29^. In 177 patients with molecular diagnosis, a lower percentage of mitochondria-related (26%) and higher percentage of non-mitochondria-related (74%) variants were detected in this cohort. This ratio was similar with a previous study^22^, wherein 37% (28/75) of patients with a definite diagnosis were in genes known to cause primary mitochondrial disease, including four mtDNA variants and 24 diagnoses in nuclear-mitochondrial genes, whereas 63% (47/75) were in non-mitochondrial genes. A precise comparison of overall diagnostic rates with previous studies is difficult, given the existence of several biases that affect the diagnostic rate, including prior mtDNA/nDNA genetic screening, population characteristics, and phenotyping accuracy. This cohort consists of patients from nine hospitals. The clinical suspicion for a mitochondrial disease of the patients varied from relatively low to very high and therefore the cohort represents the heterogeneous group of suspected mitochondrial patients. First and foremost, this is a retrospective cohort study, and it is not possible to control for factors that led to the selection of certain genetic testing methods. The diagnostic rate varied depending on the presenting clinical phenotype^30^. The highest diagnostic rates were achieved when patients with suspected MD presented with clear clinical phenotypes. It is well known that the percentage of detected MD-related genes could increase with increased MDC scores^29^. The limited diagnostic rate could be associated with relative low MDC scores in our cohort, since the diagnosis process of “genotype first” was performed widely, lacking of biochemical or histopathological supports. Median age of onset was 0.9 (0–13) years in this cohort. Genetic tests were performed rapidly and broadly in infants and children with a suspected hereditary disease^30^.

Non-mitochondrial disorders were more common than mitochondrial disorders and had features resembling mitochondrial diseases (often referred to as phenocopies). These could be broadly classified as developmental disorders with intellectual disability, metabolic disorders, myopathies, cardiomyopathies, epileptic encephalopathies, leukodystrophies, ciliopathies, amyloidosis, and other neurogenetic disorders, including basal ganglia calcification and neurodegeneration with iron accumulation. The MDC system was built by summarising known typical MDs and guiding the selection of diagnostic methods. A high MDC score is a hallmark of an MD diagnosis. Similarly in the study, there is a correlation between the degree of MD diagnosis and MDC score.

Moreover, a growing number of new nDNA and mtDNA variants have been found in patients with atypical clinical presentations or isolated symptoms. Developmental, neurological, and metabolic abnormalities are common manifestations in other metabolic diseases, leading to a high MDC score. In this cohort, most of the diagnoses were non-mitochondrial disorders, including developmental disorders, epilepsies, myopathies, and other multisystem disorders. In a previous study, 34 patients had a probable or definite mitochondrial disorder according to the modified MDC score (between 5 and 12), of these, 18% having a non-mitochondrial disorder^22^. Regarding MD mimics, combination of nDNA and mtDNA sequencing was able to detect variants in a more comprehensive perspective, thereby allowing diagnosis and differentiation of patients with other treatable conditions, enabling appropriate care and treatment for their respective diseases, while ruling out MD. As observed in our cohort, methylmalonic acidemia (*MMACHC*, *MCEE*), glycogen storage disease (*G6PC*), cerebral folate deficiency (*FOLR1*), and MDs had many overlapping features. As sequencing costs decreased and genetic sequencing became readily available, genetic tests were more acceptable than invasive muscle biopsies. After genetic tests giving a direction, these genetic metabolic diseases could be confirmed by further diagnostic tests, such as gas chromatography and mass spectrometry and liquid chromatography-tandem mass spectrometry. *SEMA3E* and *PLEC* were identified in two patients with high MDC scores. Based on the existing research, these two genes could not completely account for the symptoms observed in two patients.

The mtDNA variants had an older age of onset and higher lactate levels in this cohort. However, these were not definite guidelines for the selection of genomic tests. The distributions of MDC scores in mtDNA and mitochondria-related nDNA variants were not significantly different. Selecting mtDNA or nDNA sequences based on clinical features or MDC scores was difficult. Regarding patients with dual genomic variants, selecting candidate variants and interpreting their disease-causing roles were even more challenging. Nuclear-mitochondrial intergenomic communication disorders, which result in loss or instability of the mitochondrial genome and, in turn, impaired maintenance of qualitative and quantitative mtDNA integrity.

In the mn1 patient (MDC: 5), dual genomic variants of *MT*-*ND6* (m.14453A > G, 69.1%) and *POLG* (c.3643 + 1G > A) were tested. DNA polymerase errors have a prominent role in mtDNA mutation, arising through spontaneous errors of DNA replication or through unrepaired damage to mtDNA that introduces miscoding lesions^22^. It is already known that the *POLG* gene, responsible for the replication of mtDNA, could lead to depletion of mtDNA and/or accumulation of multiple mtDNA deletions^31^. And in a mice model with Polg mutation, the frequency of mutations was found to be 500-fold higher in heterozygous mice and 2,500-fold higher in homozygous mice than in aged wild-type mice^22^. However, regarding this patient, c.3643 + 2G > A had been reported in trans with another pathogenic variant in a child with Alpers syndrome^32^. Therefore, c.3643 + 1G > A was suspected to have an autosomal recessive inheritance. Since m.14453A > G, with a heteroplasmy of 69.1%, was a reported cause of LS and MELAS, and classified as “pathogenic”. mtDNA variant might be considered to be finally responsibe in this patient.

Guan et al. identified that mutant cell lines harbouring both m.7511A > G and *YARS2* mutations exhibited a greater deficiency in mitochondrial function^33^, and overexpression of *HARS2* in cytoplasmic hybrid cells carrying the m.12201 T > C mutation reversed mitochondrial dysfunctions^34^. It is possible that an interaction occurred between nDNA and mtDNA variants, rather than a simple single-gene disorder, since all *NARS2*, *RARS2*, *HARS2* and *EARS2* belong to a large gene family of aminoacyl tRNA synthetases that function as a translation of mtDNA. Although we tried to analyse the major causative genes between mtDNA and nDNA, some cases were still ambiguous, such as *NARS2* and *MT-TL1* (m.3243A > G) in the mn3 patient (MDC: 2). For m.3243A > G, it has been reported that patients with higher heteroplasmy levels of this point mutation exhibit Mitochondrial Encephalomyopathy, Lactic Acidosis and Stroke-Like Episodes (MELAS), while those with low heteroplasmy levels mainly have maternally inherited diabetes and deafness (MIDD). A study conducted a long-term hearing evaluation in patients with MELAS or MIDD who harbored the m.3243A > G mutation of mitochondrial DNA. They identified the age of onset of hearing loss was correlated with the heteroplasmy, and the mean age of onset of hearing loss was 28.6 years. However, hearing loss occurred earlly in our patient, and he was treated with cochlear implantation at three years old. Mutations in *NARS2* are associated with combined oxidative phosphorylation deficiency 24 and autosomal recessive deafness 94. Seizures and hearing impairments were most common in the clinical findings of *NARS2* patients^35^. A previous study reported a patient with novel *NARS2* variants, causing infantile-onset severe epilepsy. The patient had continuous bilateral clonic and myoclonic seizures and paroxysmal episodes of upward eye deviation. He had normal serum and CSF lactate levels. And initially the brain MRI was normal, repeated MR imaging showed progressive cortical and periventricular brain atrophy. However, Finsterer commented that investigations of the mDNA should be performed to tell if the *NARS2* variants altered the mtDNA sequence or resulted in mtDNA depletion. As observed in our patient, developmental regression, seizure, and hearing loss could be explaind perfectly by *NARS2* variants, however, investigations of the mDNA found another variant, m.3243A > G, which was not observed in his mother. We could not tell if m.3243A > G was caused by *NARS2* variants, and if these clinical manifestation was attributed to m.3243A > G. The mn2 patient (MDC: 4) harboured *MT*-*ND5* (m.13327A > G, 60.60%) and *RARS2* (c.1210A > G, c.622C > T). However, m.13327A > G was interpretated to be a benign variant based on the modified ACMG guidelines (BS1, BS4)^15^; *RARS2* was more likely to cause early onset epileptic encephalopathy in this patient. Similarly.

The mn4 patient (MDC: 4) harbored a mtDNA variant, m.7979G > A, and two variants in *NDUFS1* (c.1222C > T, c.61 + 3_61 + 6delGAGT). The m.7979G > A is located in *MT-CO2*, encoding a subunit of Complex IV, and *NDUFS1* encodes a Fe-S protein operating within complex I. Variants in *MT-CO2* and *NDUFS1* are associated with Leigh syndrome. The clinical manifestation of muscle weakness, growth restriction, and brain MRI with basal ganglia and brainstem involvement, can be explained by the two genes. A overlapping effect could not be comfirmed. *NDUFS1* was assessed as major pathogenic variants, however, the m.7979G > A, as a noval variant, can not be fully ruled out though at a low-level heteroplasmy.

In regard to the other two patients with mtDNA and non-mitochondria-related nDNA variants, judging whether they could be diagnosed with MDs was difficult. The patient with *MT-CYB* (m.15272A > G, 17.8%) and de novo *SMARCA2* (c.1399C > T) variants presented with psychomotor regression, seizure, dysarthria, and high serum lactate 6.81 mmol/L. Although this patient had auricular deformity, he lacked distinctive facial appearance of Nicolaides-Baraitser syndrome or Blepharophimosis-impaired intellectual development syndrome caused by *SMARCA2* variants^36^. The m.15272A > G was reclassified as “uncertain significance” according to modified ACMG^15^. *MT-CYB* is associated with Leigh syndrome, however, this patient did not harbored typical clinical features of Leigh syndrome. Existing clinical data and low-level heteroplasmy of m.15272A > G could not perfectly be connected. Another patient harboured *MT-CO3* (m.9984G > A, 16%) and *CHRNA4* (c.988G > A) variants. m.9984G > A was reported in a patient with suspected MD^37^ and reclassified as “uncertain significance” by Wong et al.^15^. *CHRNA4* is assosited with nocturnal frontal lobe epilepsy. Seizure, as the main presentation in our patient, did not provide definitive evidence.

A few cases of the coexistence of mutations in nDNA and mtDNA have been reported in a previous study. However, the lack of functional data has confused the digenic mechanisms. The relationship between nDNA and mtDNA was determined by observing attributing phenotypes, such as *KRT10* and *MT-ND6*^38^, *MT-ND1/MT-ND4* and *OPA1*, *MT-ND1/MT-ND4* and *RTN4IP1*, *ND4* and *TMEM126A*^39^. A single case could not easily distinguish the contribution of two genes, particularly both causative genes of similar MDs, as in our patients. By observing the co-segregation phenomenon and calculating the minor allele frequency of variants and prevalence of disease, a study further explored the digenic mechanisms and defined the contribution of *TRMU* and *EARS2* heterozygous variants to the clinical manifestation of the disease caused by m.14674 T > C^40^. Currently, to identify recurrence in similarly affected, unrelated patients, further studies are required to determine its likely clinical significance.

Kerr et al. found that whole-exome sequencing provided a second diagnosis in two patients who already had a pathogenic variant in mtDNA. Notably, many variants purported to be causal of disease may, in some cases, be of unknown significance or benign polymorphisms^11^. Recently, whole-genome sequencing has been recommended for early diagnosis in a patient’s local secondary or tertiary care centre and before invasive tests such as muscle biopsy^22^. At present, whole genomic sequencing is not easy to use for clinical diagnosis due to the massive amount of data it yields, and it is difficult to select candidate variants and interpret their disease-causing roles. The combination of nDNA and mtDNA sequencing is alternative for suspected MD patients.

This study had several limitations. This was a preliminary study, and the overall cohort was not followed up consistently. Variants of unknown significance in nDNA and mtDNA genes needed further tests and functional studies to verify. Our findings highlighted the importance of dual genomic sequencing for pediatric patients before widespread use of whole genome sequencing. Then, in combination with biochemical testing and muscle histology studies correctly, molecular diagnosis would be more definitive. Next, follow-up of cases and data reanalysis based on dual genomic sequencing, combined with transcriptome sequencing, could significantly improve the predictive probability of MDs and identify underlying pathogenic genes^8^.

## Conclusion

This study identified six patients with dual genomic variants in mtDNA and nDNA and demonstrated variants of non-mitochondria-related nDNA and variants of unknown significance in patients with high MDC scores. The MDC could guide the implementation of dual genomic sequencing in routine diagnostics. Esipecially, for patients assessed as“possibly having mitochondrial disorder” and “probably having mitochondrial disorder”, dual genomic sequencing provided a more comprehensive research strategy for identifiying patients with dual genomic variants and informing the risk of genetic transmission.

## Supplementary Information

Supplementary Information.

## Data Availability

The analyzed clinical and genetic data generated during the study are available from the corresponding author on reasonable request. The mtDNA and nDNA variants of patiens with mitochondrial disease are deposited in ClinVar (https://www.ncbi.nlm.nih.gov/clinvar; accession numbers: SCV002760197 - SCV002760205, SCV002761197 - SCV002761232). The raw sequencing data are not publicly available due to restriction of human data.

## References

[1] Ferreira CR, van Karnebeek C, Vockley J, Blau N (2019). A proposed nosology of inborn errors of metabolism. Genet. Med..

[2] Smeitink JA, Zeviani M, Turnbull DM, Jacobs HT (2006). Mitochondrial medicine: a metabolic perspective on the pathology of oxidative phosphorylation disorders. Cell Metab..

[3] Gibson K, Halliday JL, Kirby DM, Yaplito-Lee J, Thorburn DR, Boneh A (2008). Mitochondrial oxidative phosphorylation disorders presenting in neonates: Clinical manifestations and enzymatic and molecular diagnoses. Pediatrics.

[4] Rath S, Sharma R, Gupta R, Ast T, Chan C, Durham TJ (2021). MitoCarta3.0: an updated mitochondrial proteome now with sub-organelle localization and pathway annotations. Nucleic Acids Res..

[5] Saneto RP, Sedensky MM (2013). Mitochondrial disease in childhood: mtDNA encoded. Neurotherapeutics.

[6] Spelbrink JN, Li FY, Tiranti V, Nikali K, Yuan QP, Tariq M (2001). Human mitochondrial DNA deletions associated with mutations in the gene encoding Twinkle, a phage T7 gene 4-like protein localized in mitochondria. Nat Genet..

[7] Rahman J, Noronha A, Thiele I, Rahman S (2017). Leigh map: A novel computational diagnostic resource for mitochondrial disease. Ann. Neurol..

[8] Stenton SL, Prokisch H (2020). Genetics of mitochondrial diseases: Identifying mutations to help diagnosis. Ebiomedicine..

[9] Wortmann SB, Mayr JA, Nuoffer JM, Prokisch H, Sperl W (2017). A guideline for the diagnosis of pediatric mitochondrial disease: The value of muscle and skin biopsies in the genetics era. Neuropediatrics.

[10] Forny P, Footitt E, Davison JE, Lam A, Woodward CE, Batzios S (2021). Diagnosing mitochondrial disorders remains challenging in the omics era. Neurol Genet..

[11] Kerr M, Hume S, Omar F, Koo D, Barnes H, Khan M (2020). MITO-FIND: A study in 390 patients to determine a diagnostic strategy for mitochondrial disease. Mol. Genet. Metab..

[12] Morava E, van den Heuvel L, Hol F, de Vries MC, Hogeveen M, Rodenburg RJ (2006). Mitochondrial disease criteria: diagnostic applications in children. Neurology.

[13] Thompson K, Collier JJ, Glasgow R, Robertson FM, Pyle A, Blakely EL (2020). Recent advances in understanding the molecular genetic basis of mitochondrial disease. J. Inherit. Metab. Dis..

[14] Zhang W, Cui H, Wong LJ (2012). Comprehensive one-step molecular analyses of mitochondrial genome by massively parallel sequencing. Clin. Chem..

[15] Wong LC, Chen T, Schmitt ES, Wang J, Tang S, Landsverk M (2020). Clinical and laboratory interpretation of mitochondrial mRNA variants. Hum. Mutat..

[16] Wong LC, Chen T, Wang J, Tang S, Schmitt ES, Landsverk M (2020). Interpretation of mitochondrial tRNA variants. Genet. Med..

[17] Richards S, Aziz N, Bale S, Bick D, Das S, Gastier-Foster J (2015). Standards and guidelines for the interpretation of sequence variants: a joint consensus recommendation of the American College of Medical Genetics and Genomics and the Association for Molecular Pathology. Genet. Med..

[18] Qi Q, Jiang Y, Zhou X, Meng H, Hao N, Chang J (2020). Simultaneous Detection of CNVs and SNVs Improves the Diagnostic Yield of Fetuses with Ultrasound Anomalies and Normal Karyotypes. Genes (Basel)..

[19] Riggs ER, Andersen EF, Cherry AM, Kantarci S, Kearney H, Patel A (2020). Technical standards for the interpretation and reporting of constitutional copy-number variants: a joint consensus recommendation of the American College of Medical Genetics and Genomics (ACMG) and the Clinical Genome Resource (ClinGen). Genet. Med..

[20] Puusepp S, Reinson K, Pajusalu S, Murumets Ü, Õiglane-Shlik E, Rein R (2018). Effectiveness of whole exome sequencing in unsolved patients with a clinical suspicion of a mitochondrial disorder in Estonia. Mol. Genet. Metab. Rep..

[21] Riley LG, Cowley MJ, Gayevskiy V, Minoche AE, Puttick C, Thorburn DR (2020). The diagnostic utility of genome sequencing in a pediatric cohort with suspected mitochondrial disease. Genet. Med..

[22] Schon KR, Horvath R, Wei W, Calabrese C, Tucci A, Ibañez K (2021). Use of whole genome sequencing to determine genetic basis of suspected mitochondrial disorders: cohort study. BMJ..

[23] Wang W, Song J, Chuai Y, Chen F, Song C, Shu M (2021). The mining and construction of a knowledge base for gene-disease association in mitochondrial diseases. Sci. Rep..

[24] Graham BH (2012). Diagnostic challenges of mitochondrial disorders: complexities of two genomes. Methods Mol. Biol..

[25] Thorburn DR (2004). Mitochondrial disorders: Prevalence, myths and advances. J Inherit Metab Dis..

[26] Fang F, Liu Z, Fang H, Wu J, Shen D, Sun S (2017). The clinical and genetic characteristics in children with mitochondrial disease in China. Sci. China Life Sci..

[27] Wu T, He F, Xiao N, Han Y, Yang L, Peng J (2022). Phenotype-genotype analysis based on molecular classification in 135 children with mitochondrial disease. Pediatr. Neurol..

[28] Wortmann SB, Koolen DA, Smeitink JA, van den Heuvel L, Rodenburg RJ (2015). Whole exome sequencing of suspected mitochondrial patients in clinical practice. J. Inherit. Metab. Dis..

[29] Pronicka E, Piekutowska-Abramczuk D, Ciara E, Trubicka J, Rokicki D, Karkucińska-Więckowska A (2016). New perspective in diagnostics of mitochondrial disorders: Two years' experience with whole-exome sequencing at a national paediatric centre. J. Transl. Med..

[30] Davis RL, Kumar KR, Puttick C, Liang C, Ahmad KE, Edema-Hildebrand F (2022). Use of whole-genome sequencing for mitochondrial disease diagnosis. Neurology.

[31] Rahman S, Copeland WC (2019). POLG-related disorders and their neurological manifestations. Nat. Rev. Neurol..

[32] Mousson DCB, Chassagne M, Mayençon M, Padet S, Crehalet H, Clerc-Renaud P (2011). POLG exon 22 skipping induced by different mechanisms in two unrelated cases of Alpers syndrome. Mitochondrion.

[33] Fan W, Zheng J, Kong W, Cui L, Aishanjiang M, Yi Q (2019). Contribution of a mitochondrial tyrosyl-tRNA synthetase mutation to the phenotypic expression of the deafness-associated tRNA(Ser(UCN)) 7511A>G mutation. J. Biol. Chem..

[34] Gong S, Wang X, Meng F, Cui L, Yi Q, Zhao Q (2020). Overexpression of mitochondrial histidyl-tRNA synthetase restores mitochondrial dysfunction caused by a deafness-associated tRNA(His) mutation. J. Biol. Chem..

[35] Vafaee-Shahi M, Farhadi M, Razmara E, Morovvati S, Ghasemi S, Abedini SS (2022). Novel phenotype and genotype spectrum of NARS2 and literature review of previous mutations. Ir. J. Med. Sci..

[36] Cappuccio G, Sayou C, Tanno PL, Tisserant E, Bruel AL, Kennani SE (2020). De novo SMARCA2 variants clustered outside the helicase domain cause a new recognizable syndrome with intellectual disability and blepharophimosis distinct from Nicolaides-Baraitser syndrome. Genet. Med..

[37] Debray FG, Lambert M, Chevalier I, Robitaille Y, Decarie JC, Shoubridge EA (2007). Long-term outcome and clinical spectrum of 73 pediatric patients with mitochondrial diseases. Pediatrics.

[38] Kalinska-Bienias A, Pollak A, Kowalewski C, Lechowicz U, Stawinski P, Gergont A (2017). Coexistence of mutations in keratin 10 (KRT10) and the mitochondrial genome in a patient with ichthyosis with confetti and Leber's hereditary optic neuropathy. Am. J. Med. Genet. A..

[39] Li JK, Li W, Gao FJ, Qu SF, Hu FY, Zhang SH (2020). Mutation screening of mtDNA combined targeted exon sequencing in a cohort with suspected hereditary optic neuropathy. Transl. Vis. Sci. Technol..

[40] Hathazi D, Griffin H, Jennings MJ, Giunta M, Powell C, Pearce SF, et al. Metabolic shift underlies recovery in reversible infantile respiratory chain deficiency. *Embo J*. 2020; 39:e105364. http://doi.org/10.15252/embj.2020105364 10.15252/embj.2020105364 PMC7705457 33128823

