# Supplementary material for: Use of dual genomic sequencing to screen mitochondrial diseases in pediatrics: a retrospective analysis
Source: Sci Rep. 2023 Mar 14;13:4193. doi: 10.1038/s41598-023-31134-5 (PMC10015028; doi:10.1038/s41598-023-31134-5)
Supplement: Supplementary file 1 — Supplementary Information. [file 41598_2023_31134_MOESM1_ESM.pdf]

Use of Dual Genomic Sequencing to Screen Mitochondrial Diseases in Pediatrics: A Retrospective Analysis

Running title: Dual Genome Sequencing in Mitochondrial Diseases

Teng-Hui Wu[1], Jing Peng[1], Li Yang[1], Yan-Hui Chen[2], Xiu-Lan Lu[3], Jiao-Tian Huang[3], Jie-Yu You[4], Wen-Xian Ou-Yang[5], Yue-Yu Sun[6], Yi-Nan Xue[7], Xiao Mao[8], Hui-Ming Yan[8], Rong-Na Ren[9], Jing Xie[10], Zhi-Heng Chen[11], Victor-Wei Zhang[12, 13], Gui-Zhen Lyu[13], Fang He[1]

Supplementary table 1. Summary of genome coverage and read depth of samples

Patient ID	mtDNA sequencing		nDNA sequencing	
	genome coverage	read depth	genome coverage	read depth
n6	100.00%	7095	99.30%	118.41
n10	100.00%	5128	99.90%	116.42
n14	100.00%	6025	99.70%	133.18
n22	100.00%	5143	97.00%	93.3
n26	100.00%	5739	99.90%	93.29
n33	100.00%	6790	99.40%	145.86
n20	100.00%	5654	99.90%	117.21
n54	100.00%	6823	99.70%	275
n59	100.00%	5450	96.70%	104
n62	100.00%	6701	97.80%	168
n89	100.00%	5795	97.00%	162
n98	100.00%	5638	99.70%	93.54
n108	100.00%	5562	99.90%	343
n111	100.00%	5675	99.90%	298
n115	100.00%	6355	99.90%	212
n116	100.00%	5417	99.90%	388
n126	100.00%	5126	99.70%	119
n129	100.00%	5731	99.70%	263
n130	100.00%	5847	96.40%	116
**n155**	100.00%	6749	98.30%	106.6
n167	100.00%	7303	99.10%	391
n179	100.00%	7149	98.90%	326
n180	100.00%	5400	99.40%	328
m1	100.00%	5197	99.40%	167
m3	100.00%	5428	98.00%	98.65
m4	100.00%	4686	98.20%	143
m6	100.00%	5582	98.40%	215
m8	100.00%	5409	99.80%	153
m9	100.00%	5395	98.10%	179
m10	100.00%	5302	97.80%	121
m12	100.00%	5032	99.10%	159
m13	100.00%	8400	98.70%	170
m14	100.00%	5958	98.40%	129
m16	100.00%	5203	97.90%	221
m17	100.00%	5614	96.50%	147.99
m19	100.00%	5741	95.30%	151
mn6	100.00%	5371	92.50%	136
mn9	100.00%	7214	98.60%	178
mn1	94.50%	5503	96.40%	193.09
mn2	100.00%	6369	97.80%	88.6
mn3	100.00%	6016	98.90%	167
mn8	100.00%	5227	99.40%	329
2n4	100.00%	6379	98.60%	217
2n5	100.00%	5552	99.70%	130
n2	100.00%	5274	94.54%	112.59
n3	100.00%	6460	98.60%	108.11
n4	100.00%	5294	96.55%	131
n5	100.00%	5170	99.47%	168.66
n7	100.00%	6551	97.90%	87.05
n8	100.00%	5216	99.50%	187.5
n11	100.00%	5006	97.40%	174.76
n12	100.00%	7865	98.30%	107.69
n13	100.00%	7080	98.30%	107.18
n15	100.00%	5475	97.60%	91.98
n17	100.00%	6436	98.10%	114.09
n19	100.00%	5225	100.00%	102.86

Patient ID	mtDNA sequencing		nDNA sequencing	
	genome coverage	read depth	genome coverage	read depth
n23	100.00%	5531	99.80%	90.77
n24	100.00%	5574	99.70%	95.28
n25	100.00%	5300	98.40%	182
n27	100.00%	6580	99.40%	154
n28	100.00%	6105	98.50%	139.67
n29	100.00%	7348	97.70%	86.97
n31	100.00%	6100	98.20%	107.72
n32	100.00%	7114	97.50%	86.21
n35	100.00%	5621	93.57%	112.02
n36	100.00%	5467	96.28%	154.2
n37	100.00%	5172	98.49%	131.3
n38	100.00%	5766	95.70%	136
n39	100.00%	5315	99.60%	123.11
n40	100.00%	7390	99.50%	183
n41	100.00%	5200	92.10%	168
n43	100.00%	5271	60.90%	113
n46	100.00%	5445	98.10%	158
n47	100.00%	5120	92.90%	128
n48	100.00%	5324	97.90%	168
n50	100.00%	5196	97.70%	336
n51	100.00%	5241	97.50%	151
n52	100.00%	5849	97.70%	142
n55	100.00%	5964	96.60%	131
n57	100.00%	5945	97.80%	157
n58	100.00%	5477	99.20%	198
n61	100.00%	6193	99.50%	202
n63	100.00%	5497	98.30%	357
n66	100.00%	6292	94.00%	112
n67	100.00%	5863	96.10%	156
n68	100.00%	6231	96.00%	130
n69	100.00%	5292	97.80%	178
n70	100.00%	5405	97.80%	152
n71	100.00%	5471	97.20%	160
n72	100.00%	9455	98.20%	164
n73	100.00%	5212	97.20%	155
n74	100.00%	5255	96.20%	144
n75	100.00%	5487	97.00%	156
n76	100.00%	5567	96.40%	127
n77	100.00%	6123	99.50%	393
n78	100.00%	5145	97.40%	168
n80	100.00%	5196	94.30%	121

Patient ID	mtDNA sequencing		nDNA sequencing	
	genome coverage	read depth	genome coverage	read depth
n81	100.00%	5216	96.10%	133
n83	100.00%	5812	97.50%	155
n84	100.00%	5715	94.30%	113
n85	100.00%	7544	95.40%	114
n86	100.00%	5901	97.30%	180
n87	100.00%	5813	94.70%	112
n88	100.00%	5542	97.20%	146
n91	100.00%	5588	95.00%	146
n92	100.00%	7387	96.90%	212
n93	100.00%	5350	96.10%	135
n94	100.00%	5130	93.71%	133.57
n95	100.00%	6883	93.16%	135.83
n96	100.00%	5594	95.77%	135.83
n99	100.00%	5072	98.20%	135.83
n100	100.00%	5419	95.82%	117.87
n101	100.00%	5776	98.00%	176
n103	100.00%	5091	97.80%	159
n105	100.00%	5582	96.41%	153.42
n106	100.00%	7299	97.80%	130.56
n107	100.00%	5264	96.43%	141.08
n109	100.00%	5734	99.50%	295
n110	100.00%	5818	99.20%	283
n112	100.00%	5667	99.10%	169
n113	100.00%	5522	99.60%	117
n117	100.00%	7403	99.90%	346
n118	100.00%	5706	99.70%	324
n119	100.00%	6990	99.50%	337
n120	100.00%	7357	99.90%	279
n121	100.00%	7430	99.90%	7265
n123	100.00%	5724	97.50%	185
n124	100.00%	5643	96.70%	169
n125	100.00%	7459	97.40%	132
n127	100.00%	5808	97.80%	159
n128	100.00%	5817	96.90%	115
n131	100.00%	5688	97.40%	140
n132	100.00%	7364	97.50%	142
n133	100.00%	5664	97.20%	126
n134	100.00%	5672	96.40%	183
n135	100.00%	5363	99.70%	348
n136	100.00%	5873	96.70%	109
n137	100.00%	6773	96.53%	138.49

Patient ID	mtDNA sequencing		nDNA sequencing	
	genome coverage	read depth	genome coverage	read depth
n138	100.00%	5748	98.70%	109
n139	100.00%	5564	96.70%	134
n157	100.00%	5609	99.30%	126
n158	100.00%	7043	96.40%	212
n159	100.00%	4507	97.80%	156
n160	100.00%	7034	98.80%	124
n161	100.00%	6087	95.80%	153
n163	100.00%	5309	96.70%	137
n164	100.00%	6150	96.80%	164
n168	100.00%	6038	97.20%	98
n169	100.00%	5206	97.80%	175
n170	100.00%	5591	94.30%	208
n171	100.00%	5308	98.20%	211
n172	100.00%	5479	98.00%	178
n173	100.00%	6177	97.80%	167
n174	100.00%	6122	98.50%	123
n175	100.00%	5930	95.30%	143
n176	100.00%	5303	99.80%	212
n177	100.00%	5310	99.43%	153
n178	100.00%	6280	98.30%	134
n181	100.00%	5839	97.40%	164
n182	100.00%	5600	92.82%	109.47
n183	100.00%	5722	96.60%	167
n184	100.00%	6682	93.77%	110.44
n185	100.00%	5534	96.20%	136
n186	100.00%	5321	94.90%	159
2n1	100.00%	5507	95.80%	163
2n2	100.00%	5425	98.30%	189
2n3	100.00%	5563	98.00%	171
2n6	100.00%	5400	98.50%	201
2n13	100.00%	5150	97.90%	98
2n14	100.00%	5889	97.80%	118
mn4	100.00%	5805	95.30%	123
mn5	100.00%	5371	97.20%	110
mn10	100.00%	5715	99.40%	131
n114	100.00%	7489	99.80%	379
n156	100.00%	6789	97.90%	132
n162	100.00%	5793	99.20%	153
u1	100.00%	5324	99.57%	172
u2	100.00%	5333	90.90%	71
u3	100.00%	5539	98.70%	125

Patient ID	mtDNA sequencing		nDNA sequencing	
	genome coverage	read depth	genome coverage	read depth
u4	100.00%	6933	99.00%	121
u5	100.00%	5285	97.90%	89
u6	100.00%	6849	99.00%	276
u7	100.00%	7061	99.00%	108
u8	100.00%	5809	98.90%	143
u9	100.00%	5291	99.43%	166
u10	100.00%	5538	99.64%	167
u11	100.00%	7419	97.50%	81
u12	100.00%	4752	96.30%	68
u13	100.00%	5300	98.90%	129
u14	100.00%	4689	98.90%	135
u15	100.00%	5373	97.50%	164
u16	100.00%	6360	99.50%	165
u17	100.00%	5168	96.90%	80
u18	100.00%	5824	96.20%	135
u19	100.00%	5105	98.70%	118
u20	100.00%	4935	97.80%	81
u21	100.00%	6937	98.30%	103
u22	100.00%	5056	97.80%	156
u23	100.00%	5591	98.38%	120
u24	100.00%	5296	98.22%	124
u25	100.00%	6834	99.30%	135
u26	100.00%	5599	95.06%	112
u27	100.00%	5815	99.10%	115
u28	100.00%	6905	98.60%	135
u29	100.00%	5373	99.50%	134
u30	100.00%	5019	99.58%	186
u31	100.00%	6983	99.00%	181
u32	100.00%	7111	97.30%	208
u33	100.00%	5610	98.60%	137
u34	100.00%	7521	97.60%	81
u35	100.00%	5297	96.50%	112
u36	100.00%	5317	97.01%	140
u37	100.00%	6985	93.70%	138
u38	100.00%	5150	95.10%	95
u39	100.00%	5856	97.10%	78.94
u40	100.00%	7165	97.80%	135
u41	100.00%	5160	97.01%	171
u42	100.00%	6332	97.80%	91
u43	100.00%	5075	98.00%	146
u44	100.00%	7579	98.40%	79.21

Patient ID	mtDNA sequencing		nDNA sequencing	
	genome coverage	read depth	genome coverage	read depth
u45	100.00%	7356	95.30%	71
u46	100.00%	5131	98.50%	124
u47	100.00%	7729	98.40%	108
u48	100.00%	5083	98.01%	205
u49	100.00%	6946	98.00%	96
u50	100.00%	7705	98.29%	113
u51	100.00%	5140	94.60%	87
u52	100.00%	6374	99.45%	135
u53	100.00%	5515	99.10%	131
u54	100.00%	5169	97.80%	195
u55	100.00%	6470	97.80%	164
u56	100.00%	5454	95.70%	149
u57	100.00%	7541	97.90%	123
u58	100.00%	7329	97.60%	96
u59	100.00%	6738	98.80%	271
u60	100.00%	5495	95.80%	116
u61	100.00%	6758	98.60%	92
u62	100.00%	6500	98.40%	93
u63	100.00%	5807	98.70%	121
u64	100.00%	5770	99.45%	156
u65	100.00%	6062	98.70%	103
u66	100.00%	7172	96.90%	272
u67	100.00%	6464	98.20%	98
u68	100.00%	9849	97.20%	135
u69	100.00%	5800	99.44%	152
u70	100.00%	6531	98.90%	131
u71	100.00%	6220	94.75%	111
u72	100.00%	5496	97.50%	174
u73	100.00%	9341	97.90%	104
u74	100.00%	5158	92.20%	138
u75	100.00%	6179	98.61%	142
u76	100.00%	5522	99.50%	165
u77	100.00%	5419	96.39%	148
u78	100.00%	5313	95.87%	126
u79	100.00%	7252	96.40%	152
u80	100.00%	5240	99.60%	99
u81	100.00%	6183	94.15%	139
u82	100.00%	5956	98.20%	174
u83	100.00%	5971	93.50%	120
u84	100.00%	5291	98.29%	136
u85	100.00%	5438	99.45%	173

Patient ID	mtDNA sequencing		nDNA sequencing	
	genome coverage	read depth	genome coverage	read depth
u86	100.00%	5336	99.70%	128
u87	100.00%	5539	99.60%	116
u88	100.00%	5289	97.40%	131
u89	100.00%	6380	99.20%	117
u90	100.00%	5521	96.43%	154
u91	100.00%	5481	97.40%	138
u92	100.00%	5090	96.50%	119
u93	100.00%	7221	97.80%	139
u94	100.00%	5260	97.60%	166
u95	100.00%	7316	99.90%	193
u96	100.00%	5868	96.00%	110
u97	100.00%	5457	96.40%	119
u98	100.00%	5348	97.30%	192
u99	100.00%	5416	97.60%	162
u100	100.00%	5869	95.40%	217
u101	100.00%	5093	97.30%	162
u102	100.00%	5413	96.90%	139
u103	100.00%	5871	97.10%	149
u104	100.00%	6940	98.90%	95
u105	100.00%	5461	93.70%	161
u106	100.00%	5484	99.60%	115
u107	100.00%	9618	97.90%	168
u108	100.00%	7629	98.20%	186
u109	100.00%	5884	99.90%	147
u110	100.00%	5346	97.50%	259
u111	100.00%	5538	99.90%	137
u112	100.00%	5361	98.90%	169
u113	100.00%	5274	99.70%	234
u114	100.00%	5071	97.60%	161
u115	100.00%	5416	99.90%	195
u116	100.00%	5764	97.60%	173
u117	100.00%	5102	96.40%	134
u118	100.00%	5633	98.30%	162
u119	100.00%	5100	97.60%	149
u120	100.00%	5194	98.70%	176
u121	100.00%	5281	97.00%	132
u122	100.00%	5975	97.80%	218
u123	100.00%	6926	98.00%	153
u124	100.00%	5300	95.80%	106
u125	100.00%	5438	98.10%	160
u126	100.00%	7901	93.70%	311

Patient ID	mtDNA sequencing		nDNA sequencing	
	**genome coverage**	**read depth**	**genome coverage**	**read depth**
u127	100.00%	5964	98.40%	110
u128	100.00%	5111	95.80%	136
u129	100.00%	6045	97.40%	167
u130	100.00%	5162	98.20%	158
u131	100.00%	5460	97.70%	306
u132	100.00%	6345	97.90%	152
u133	100.00%	5110	97.70%	178
u134	100.00%	5417	96.60%	120
u135	100.00%	5417	99.20%	165
u136	100.00%	5361	97.60%	163
u137	100.00%	5060	97.50%	98
u138	100.00%	6409	96.50%	165
u139	100.00%	5660	99.10%	152
u140	100.00%	5150	97.20%	116
u141	100.00%	5438	97.40%	180
u142	100.00%	5725	96.00%	140
u143	100.00%	5803	94.80%	212
u144	100.00%	5690	99.10%	114
u145	100.00%	5585	96.50%	156
u146	100.00%	5533	97.90%	139
u147	100.00%	5244	97.00%	142
u148	100.00%	5437	98.50%	121
u149	100.00%	5230	99.40%	305
u150	100.00%	5480	99.70%	158
u151	100.00%	5692	99.80%	136
u152	100.00%	5849	98.40%	182
u153	100.00%	5540	95.90%	278
u154	100.00%	5891	99.50%	315
u155	100.00%	5462	97.50%	139
u156	100.00%	5072	97.10%	154
u157	100.00%	5974	96.20%	124
u158	100.00%	5465	97.40%	330
u159	100.00%	6416	97.40%	209
u160	100.00%	6251	99.60%	160
u161	100.00%	6848	98.70%	135
u162	100.00%	6008	94.30%	134
u163	100.00%	5462	96.20%	129
u164	100.00%	5967	98.00%	99
u165	100.00%	6707	99.40%	173
u166	100.00%	5783	98.90%	138
u167	100.00%	5512	97.70%	193

Patient ID	mtDNA sequencing		nDNA sequencing	
	**genome coverage**	**read depth**	**genome coverage**	**read depth**
u168	100.00%	5683	95.80%	158
u169	100.00%	5712	97.90%	164
u170	100.00%	5425	96.70%	175
u171	100.00%	5629	97.20%	130
u172	100.00%	9080	96.70%	134
u173	100.00%	5697	91.20%	95
u174	100.00%	5712	97.80%	294
u175	100.00%	5634	97.62%	108
u176	100.00%	6528	97.70%	185
u177	100.00%	5632	94.80%	138
u178	100.00%	6442	98.90%	110
u179	100.00%	5473	93.30%	144
u180	100.00%	6351	99.30%	145
u181	100.00%	5160	98.50%	181
u182	100.00%	7341	98.30%	246
u183	100.00%	7540	99.00%	122
u184	100.00%	6489	99.70%	185
u185	100.00%	5682	88.60%	137
u186	100.00%	7539	98.80%	138
u187	100.00%	7050	98.20%	93
u188	100.00%	6098	99.10%	129
u189	100.00%	6394	97.20%	76
u190	100.00%	6070	98.70%	119
u191	100.00%	7049	95.80%	213
u192	100.00%	5300	99.70%	282
u193	100.00%	6716	99.70%	183
u194	100.00%	5779	95.30%	110
u195	100.00%	5726	93.50%	93
u196	100.00%	7511	97.60%	317
u197	100.00%	7237	94.00%	162
u198	100.00%	7104	99.80%	296
u199	100.00%	7546	95.40%	277
u200	100.00%	7104	99.80%	204
u201	100.00%	5935	98.40%	212
u202	100.00%	5706	96.50%	118
u203	100.00%	5690	95.20%	112
u204	100.00%	5749	93.50%	137
u205	100.00%	5337	96.70%	113
u206	100.00%	5091	97.90%	197
u207	100.00%	5794	97.80%	205
u208	100.00%	5684	98.30%	124

Patient ID	mtDNA sequencing		nDNA sequencing	
	genome coverage	read depth	genome coverage	read depth
u209	100.00%	5142	97.40%	131
u210	100.00%	5814	97.80%	163
u211	100.00%	5747	97.40%	146
u212	100.00%	7354	96.70%	119
u213	100.00%	5693	97.80%	175
u214	100.00%	7282	98.10%	108
u215	100.00%	7442	98.30%	132
u216	100.00%	7388	94.45%	110
u217	100.00%	5063	95.37%	122
u218	100.00%	5588	95.81%	123
u219	100.00%	5820	96.17%	133
u220	100.00%	5786	96.08%	123
u221	100.00%	6470	97.00%	141
u222	100.00%	5758	97.80%	196
u223	100.00%	5710	97.12%	151
u224	100.00%	5628	95.58%	130
u225	100.00%	5657	97.90%	228
u226	100.00%	5483	98.40%	158
u227	100.00%	6359	97.50%	96
u228	100.00%	5388	97.40%	231
u229	100.00%	5684	95.90%	194
u230	100.00%	6386	97.40%	148
u231	100.00%	5835	98.60%	127
u232	100.00%	6488	98.40%	271
u233	100.00%	6356	99.00%	167
u234	100.00%	5735	99.40%	119
u235	100.00%	7356	98.40%	134
u236	100.00%	6573	97.90%	128
u237	100.00%	5835	97.60%	122
u238	100.00%	6868	96.40%	163
u239	100.00%	6848	97.10%	173
u240	100.00%	5388	98.60%	137
u241	100.00%	5365	98.00%	164
u242	100.00%	5729	96.80%	138
u243	100.00%	5396	98.30%	174
u244	100.00%	5734	97.20%	146
u245	100.00%	5753	98.20%	185
u246	100.00%	7422	98.00%	136
u247	100.00%	6468	97.80%	185
u248	100.00%	5738	97.40%	136
u249	100.00%	5238	97.40%	174

Patient ID	mtDNA sequencing		nDNA sequencing	
	genome coverage	read depth	genome coverage	read depth
u250	100.00%	6346	95.37%	136
u251	100.00%	6738	96.17%	185
u252	100.00%	5657	97.30%	135
u253	100.00%	5735	95.70%	174
u254	100.00%	5787	98.20%	136
u255	100.00%	5295	96.20%	186
u256	100.00%	5786	98.20%	130
u257	100.00%	7462	94.00%	164
u258	100.00%	6868	96.70%	135
u259	100.00%	5768	98.60%	105
u260	100.00%	5683	97.80%	157
u261	100.00%	7107	97.20%	146
u262	100.00%	6489	94.70%	201
u263	100.00%	6352	96.30%	96
u264	100.00%	6584	94.00%	216
u265	100.00%	5572	96.90%	136
u266	100.00%	5437	98.60%	138
u267	100.00%	6365	94.30%	185
u268	100.00%	5469	96.80%	136
u269	100.00%	5011	95.10%	103
u270	100.00%	5256	94.80%	138
u271	100.00%	5343	95.70%	123
u272	100.00%	5743	92.00%	119
u273	100.00%	5142	92.00%	120
u274	100.00%	6433	98.20%	97
u275	100.00%	6260	97.80%	110
u276	100.00%	5512	98.00%	333
u277	100.00%	5537	96.20%	111
u278	100.00%	5045	95.93%	139
u279	100.00%	5623	99.60%	317
u280	100.00%	7050	98.20%	9
u281	100.00%	6745	99.40%	175
u282	100.00%	5090	96.30%	140
u283	100.00%	7049	98.90%	137
u284	100.00%	5799	95.60%	158
u285	100.00%	5233	94.00%	162
u286	100.00%	9470	98.40%	367
u287	100.00%	5358	99.80%	148
u288	100.00%	5325	96.90%	142
u289	100.00%	5776	97.60%	199
u290	100.00%	5170	93.00%	149

Patient ID	mtDNA sequencing		nDNA sequencing	
	genome coverage	read depth	genome coverage	read depth
u291	100.00%	5376	98.60%	149
u292	100.00%	5423	96.80%	168
u293	100.00%	5476	99.50%	131
u294	100.00%	5787	94.30%	276
u295	100.00%	7552	99.30%	132
u296	100.00%	5084	98.60%	176
u297	100.00%	7063	98.50%	103
u298	100.00%	5581	97.80%	158
u299	100.00%	9662	97.80%	159
u300	100.00%	5646	96.30%	155
u301	100.00%	5636	95.20%	142
u302	100.00%	5212	97.20%	181
u303	100.00%	5241	96.30%	71
u304	100.00%	5490	96.00%	117
u305	100.00%	5175	93.40%	166
u306	100.00%	5420	94.70%	166
u307	100.00%	5276	99.50%	250
u308	100.00%	5367	96.80%	182
u309	100.00%	6872	98.80%	124

Patient ID	mtDNA sequencing		nDNA sequencing	
	genome coverage	read depth	genome coverage	read depth
u310	100.00%	5735	132.00%	99
u311	100.00%	6884	98.90%	102
u312	100.00%	5635	92.10%	105
u313	100.00%	5311	98.50%	126
u314	100.00%	5494	91.30%	145
u315	100.00%	5869	96.80%	137
u316	100.00%	5579	165.00%	98
u317	100.00%	7801	97.20%	119
u318	100.00%	5040	96.10%	72
u319	100.00%	6316	95.90%	258
u320	100.00%	6163	95.80%	117
u321	100.00%	6405	95.30%	117
u322	100.00%	5788	92.30%	135
u323	100.00%	7258	98.40%	105
u324	100.00%	5320	97.50%	176
u325	100.00%	5785	97.80%	95
u326	100.00%	5655	99.60%	324
u327	100.00%	6724	99.70%	124

Supplemental table 2. Summary of clinical features of patients with non-mitochondria-related variants.

Patient	Gender	Gene	Inheritance pattern	Variant	Zygosity	Origin	Age of onset (years)	Clinical features	Serum lactate (mmol/L)	MRI	ACMG	Evidence	Final clinical diagnosis	Novel variant
n2	F	*SCN8A*	AD	c.1099A>G (p.M367V)	het	de novo	0.4	Seizure	2.68	cerebellar atrophy	LP	PM1+PM2+PP3+PP5	Epileptic encephalopathy	N
n3	M	*STXBP1*	AD	c.1439C>T (p.P480L)	het	de novo	0.8	DD, myoclonus	2.55	normal	P	PS2+PM1+PM2+PP2+PP3	Epileptic encephalopathy	N
n4	F	*EEF1A2*	AD	c.1331_1333del (p.K444del)	het	de novo	0.5	Seizure, nystagmus	Na	cerebellar atrophy	LP	PS2+PM2+PM4	GDD, epilepsy	Y
n5	F	*MECP2*	XLD	c.808C>T (p.R270*)	het	de novo	4.0	Development regression	Na	Na	P	PVS1+PS2+PM2	Rett syndrome	Y
n7	M	*USH2A*	AR	c.10931C>T (p.T3644M); c.7616C>T (p.P2539L); c.4492C>G (p.P1498A)	com.het	parental	0.3	DD, impaired hearing, cataract	3.75	cerebellar dysplasia	VUS/VUS/VUS	PM2+BP4/PP3/PM2	GDD, deafness	N/N/N
n8	M	*POMT1*	AR	c.824+1G>A; c.1777G>A (p.A593T)	com.het	parental	0.3	DD, abnormal electromyography	2.8	normal	LP/P	PVS1+PM3+PP3/PS1+PS4+PM2+PP3	Muscular dystrophy-dystroglycanopathy (limb-girdle) type C1	Y/Y
n11	M	*JAG1*	AD	c.2698C>T (p.R900*)	het	de novo	1.5	Patent foramen ovale, DD, seizure	6.64	cortical atrophy	P	PVS1+PS1+PS2+PM2+PP3	Tetralogy of Fallot	N
n12	F	*UBE3A*	AD	c.2507_2510delAAGA (p.K836Rfs*4)	het	maternal	0.3	DD, seizure, strabismus	2	normal	LP	PM2+PM4+PM6	Angelman syndrome	Y
n13	F	*ASL*	AR	c.331C>T (p.R111W); c.434A>G (p.D145G)	com.het	parental	1.3	Seizure, elevated AST	3.5	white matter abnormalities	P/P	PS1+PS4+PM2/PS1+PS4+PM2	Argininosuccinic aciduria	N/N
n15	F	*GNE*	AD/AR	c.864T>A (p.H288Q)	het	de novo	0.5	DD, seizure, strabismus	Na	white matter abnormalities	LP	PS2+PM2	GDD, epilepsy	Y
n17	M	*ARSA*	AR	c.464A>G (p.Q155R); c.1222A>C (p.S408R)	com.het	parental	1.4	DD, elevated CK	3.1	white matter abnormalities	VUS/VUS	PM2+PP3/PM2+PP3	GDD	N/Y
n23	F	*BCORL1*	XLR	c.1891C>T (p.Q631*)	het	de novo	0.3	Seizure, multi-malformations	3.4	hydrocephalus	LP	PS2+PM2+PP3	Shukla-Vernon syndrome	Y
n24	F	*MECP2*	XLD	c.880C>T (p.R294*)	het	de novo	0.7	DD, gastrointestinal disorder, hypothyroidism	2.5	normal	P	PVS1+PS2+PM2	Rett syndrome	Y
n27	F	*AIMP1*	AR	c.191_192delAA (p.Q64Rfs*25); c.479delG (p.G160Vfs*4)	com.het	parental	at birth	DD, feeding difficulties, hearing loss, microcephalus	3.51	cortical atrophy	LP/LP	PVS1+PM2/PVS1+PM2	Hypomyelinating leukodystrophy-3	Y/Y
n28	F	*GRIN2B*	AD	c.1661T>C (p.F554S)	het	de novo	at birth	DD, feeding difficulties, genital malformation	Na	normal	LP	PS2+PM2+PP3	Developmental and epileptic encephalopathy 27	N
n29	F	*G6PC*	AR	c.226A>C (p.K76Q); c.648G>T（p.L216L）	com.het	parental	at birth	Growth restriction, hypoglycemia, liver disorder, hyperlipidemia	6.3	white matter abnormalities	VUS/P	PM2+PP3/PVS1+PS3+PM2+PP3	Glycogen storage disease due to glucose-6-phosphatase deficiency type IA	N/N
n31	M	*HUWE1*	XL	c.693+1G>T	het	de novo	1.0	DD	Na	normal	P	PVS1+PS2+PM2	Intellectual developmental disorder, X-linked syndromic, Turner type	Y
n32	F	*TUBA4A*	AD	c.679C>T (p.L227F)	het	de novo	0.3	DD, hypotonia, abnormal electromyography	3.37	Na	LP	PS2+PM2+PP3	Amyotrophic lateral sclerosis-22	Y
n36	F	*CSNK2B*	AD	c.462_465del (p.D155Afs*70)	het	de novo	0.3	Seizure	2.89	slight widening of the anterior sulcus fissure pool and supratentorial ventricles	P	PVS1+PS2+PM2+PP3	Poirier-Bienvenu neurodevelopmental syndrome	Y
n37	M	*MYH7*	AD	c.2146G>A (p.G716R)	het	de novo	11.7	Cardiac arrest	2.54	normal	P	PS2+PS4+PM1+PM2+PP1	Sudden cardiac arrest	N
n38	F	*SCN1A*	AD	c.4853-2A>C	het	maternal	0.5	Seizure	4.37	cyst	P	PVS1+PM2+PP3	Dravet syndrome	N

Patient	Gender	Gene	Inheritance pattern	Variant	Zygosity	Origin	Age of onset (years)	Clinical features	Serum lactate (mmol/L)	MRI	ACMG	Evidence	Final clinical diagnosis	Novel variant
n39	F	*GRID2*	AR	c.1241G>A (p.R414Q); c.1468G>C (p.V490L)	com.het	parental	0.5	Motor delay	1.55	cerebellar atrophy	VUS/VUS	PM2+PP3/PM2+PP3	Motor delay	Y/Y
n40	M	*GRIN2B*	AD	c.2461G>T (p.V821F)	het	de novo	0.3	DD	3.1	normal	LP	PS2+PM2_P+PP3	Developmental and epileptic encephalopathy 27	N
n41	M	*SCN8A*	AD	c.5615G>A (p.R1872Q)	het	de novo	0.3	Seizure, development regression	3.62	cerebral hypoplasia with bilateral widening of the lateral fissure	P	PS2_VS+PS3+ PM2_P+PM5	Developmental and epileptic encephalopathy	N
n43	M	*SCN1A*	AD	c.2867T>A (p.M956K)	het	de novo	0.3	Seizure, elevated AST	4.03	cortical abnormalities	P	PS2+PS3+PM2_P	Dravet syndrome	N
n46	M	*SPTBN2*	AR /AD	c.1307T>C (p.M436T)	het	de novo	1.0	DD	4.39	cerebellar atrophy	LP	PS2+PM2_P+PP3	Spinocerebellar ataxia	Y
n47	M	*FOLR1*	AR	c.524G>T (p.C175F)	hom	parental	2.0	Growth restriction, seizure, microcephalus, ataxia	5.47	cerebellar atrophy	VUS	PM2_P+PP3	Cerebral folate transport deficiency	N
n50	F	*AKT3*	AD	c.803T>C (p.V268A)	het	de novo	at birth	Macrocephaly, hypotonia	1.24	cortical abnormalities	LP	PS2+ PM2_P+ PP3	Megalencephaly-polymicrogyria-polydactyly-hydrocephalus syndrome 2	N
n51	F	*EIF2B2*	AR	c.547C>T (p.R183*); c.254T>A (p.V85E)	com.het	parental	0.6	DD, seizure, strabismus	4.11	white matter abnormalities	P/LP	PVS1+PM3+PM2_P/ PS3_M+PM3_S+PM2_P	Leukoencephalopathy	N/N
n52	M	*NPC1*	AR	c.3425T>C (p.M1142T); c.1552C>T (p.R518W)	com.het	parental	12.0	Muscle weakness, development regression	3.28	normal	LP/LP	PM3_S+PP3+PM2_P/ PM3_S+PP3+PM2_P+PM5	Niemann-Pick disease	N/N
n55	F	*ATP1A2*	AD	c.1126A>G (p.T376A)	het	de novo	8.0	Paroxysmal loss of consciousness, muscle weakness	1.8	normal	LP	PS2+PP3+PM2_P+PM5	Alternating hemiplegia of childhood	Y
n57	M	*CACNA1A*	AD	c.2101G>A (p.G701R)	het	de novo	infancy	DD	2.84	cerebellar atrophy	LP	PS2+PP3+PM2_P	Spinocerebellar Ataxia	Y
n58	M	*SGCE*	AD	c.282C>G (p.Y94*)	het	de novo	1.6	motor impairment, myoclonus	23.7	normal	P	PVS1+PS2+PM2_P	Myoclonus-dystonia syndrome	Y
n61	F	*TTN*	AR/AD	c.35341G>A (p.G11781S); c.36577+4A>G; c.41856dupA (p.Y13953Ifs*8)	com.het	parental	0.3	Motor delay, muscle weakness	4.06	normal	VUS/VUS /LP	PM2_P/ PM2_P+PP3/ PVS1+PM2_P	Limb-girdle muscular dystrophy-10	Y/Y/Y
n63	M	*PIGA*	XLR	c.98A>G (p.H33R)	hem	maternal	0.8	Seizure, development regression	Na	normal	VUS	PP3+PM2_P	Epileptic encephalopathy	N
n66	F	*CIC*	AD	c.2644C>T (p.P882S)	het	de novo	12.0	ID, impaired vision	1.99	normal	VUS	PS2_M+PM2_P	Intellectual developmental disorder	N
n67	F	*LAMA2*	AR	c.595T>C (p.C199R); c.3283C>T (p.R1095*)	com.het	parental	1.0	Muscle weakness, gastrocnemius hypertrophy, abnormal electromyography	1.2	Na	LP/P	PM2_P+PP3+PM3+PM5/ PVS1+PM3+PM2_P	Muscular dystrophy, limb-girdle, autosomal recessive 23	N/N
n68	F	*SMC1A*	XLD	c.3151C>T (p.R1051*)	het	de novo	3.0	Seizure	1.35	cerebellar dysplasia	P	PVS1+PM2_P+PS2_M	Developmental and epileptic encephalopathy	N
n69	M	*CEP290*	AR	c.5972delA (p.K1991Rfs*6)	hom	parental	0.3	DD, strabismus, nystagmus	11.42	cortical atrophy	P	PVS1+PM2_P+PM3_P	Joubert syndrome 5	N
n70	M	*PRRT2*	AD	c.860C>A (p.A287D)	het	paternal	0.3	Seizure	3.24	thin corpus callosum	VUS	PM2_P+PP3	Benign familial infantile seizures-2	Y
n71	M	*GCH1*	AR/AD	c.604G>A (p.V202I)	hom	parental	1.0	Dystonia	0.87	normal	LP	PM2_P+PP3+PM3+PM1_P+PP4	Dopa-Responsive Dystonia	N

Patient	Gender	Gene	Inheritance pattern	Variant	Zygosity	Origin	Age of onset (years)	Clinical features	Serum lactate (mmol/L)	MRI	ACMG	Evidence	Final clinical diagnosis	Novel variant
n73	F	*KCNQ2*	AD	c.997C>T (p.R333W)	het	de novo	at birth	Seizure	2.58	cyst	P	PS2+PS3_P+PM2_P+PP3+PM1_P+PM5	Developmental and epileptic encephalopathy 7	N
n74	F	*GRIN2B*	AD	c.2459G>T (p.G820V)	het	de novo	infancy	Infantile spasms, congenital heart disease	1.26	normal	P	PS2+PM2_P+PP3+PM5_S	Developmental and epileptic encephalopathy 27	N
n75	M	*POLR2A*	AD	c.5623T>C (p.S1875P)	het	de novo	1.0	Speech delay	1.46	Bilateral paraventricular, cerebellum, right basal ganglia, bilateral insula hyperintensity	VUS	PM2_P+PP3	DD	Y
n76	M	*DYNC1H1*	AD	c.3185A>G (p.D1062G)	het	de novo	2.0	DD, ADHD, dystonia	5	normal	LP	PS2+PM2_P+PP3	Intellectual developmental disorder	Y
n77	F	*COL2A1*	AD	c.2887G>T (p.G963C)	het	de novo	0.3	DD, severe pneumonia, cleft palate, laryngeal chondrodysplasias	2.48	Na	LP	PS2+PM2_P+PP3+PM1	DD, chondrodysplasias	N
n78	F	*SCN8A*	AD	c.2929C>G (p.L977V)	het	de novo	0.3	Seizure	1.18	normal	LP	PS2+PM2_P+PP3	Developmental and epileptic encephalopathy 13	N
n80	M	*SLC6A1*	AD	c.718G>T (p.V240F)	het	de novo	3.0	Speech delay, atonic seizure	1.69	normal	LP	PS2+PM2_P+PP3	Epileptic encephalopathy	N
n81	M	*SPTAN1*	AD	c.6749T>C (p.L2250P)	het	de novo	at birth	DD	1.47	cerebellar atrophy	LP	PS2+PM2_P+PP3	ID, cerebellar atrophy	N
n83	M	*KANSL1*	AD	c.868C>T (p.R290*)	het	de novo	at birth	DD, hypotonia	1.35	normal	P	PVS1+PS2+PM2_P	DD, hypotonia	N
n84	F	*KCNQ2*	AD	c.297-2A>C	het	maternal	infancy	Seizure, liver disorder	6.47	normal	LP	PVS1+PM2_P	Benign Familial Neonatal Seizures	N
n85	F	*GRIN2B*	AD	c.1946A>G (p.N649S)	het	de novo	0.3	DD, hypotonia	1.35	normal	LP	PS2+PM2_P+PP3	GDD	N
n86	M	*SGSH*	AR	c.1130G>A (p.R377H); c.1063G>A (p.E355K)	com.het	parental	5.0	DD	1.04	white matter abnormalities	P/P	PM2_P+PP3+PM3_VS/ PM2_P+PP3+PM3_VSt	Mucopolysaccharidosis type IIIA	N/N
n87	M	*ATM*	AR	c.8283_8284del(p.Q2762Afs*6); c.5170G>T (p.E1724*)	com.het	parental	0.3	DD, development regression, growth restriction	3.28	cerebellar atrophy	P/P	PM2_P+PVS1+PM3+PP4/ PM2_P+PVS1+PP1_M	Ataxia-telangiectasia	Y/Y
n88	M	*SYNGAP1*	AD	c.490C>T (p.R164*)	het	de novo	0.6	DD, myoclonus	2.71	Na	P	PM2_P+PVS1+PS2_M	Intellectual developmental disorder	N
n91	F	*SLC2A1*	AD /AR	c.481C>T (p.Q161*)	het	de novo	1.0	Seizure, motor delay	1.61	normal	P	PM2_P+PVS1+PS2	GLUT1 deficiency syndrome 1	N
n92	M	*CACNA1A*	AD	c.4930G > A (D160N)	het	de novo	0.6	DD, microcephalus	4.58	cerebellar atrophy	LP	PS2+PM2+PP3	Developmental and epileptic encephalopathy	Y
n93	F	*RHOBTB2*	AD	c.359G>A (p.G120E)	het	de novo	0.3	DD, seizure	1.19	cortical abnormalities	LP	PS2+PM2+PP3	Developmental and epileptic encephalopathy 64	Y
n94	F	*VAC14*	AR	c.2042G>A (p.R681H); c.215G>A (p.G72D)	com.het	parental	1.0	DD, dystonia, liver disorder	1.79	normal	VUS/VUS	PM2+PP3/PM2+PP3	Dystonia	N/Y
n95	M	*CHRNE*	AR/AD	c.1168C>G (p.R390G)	het	de novo	6.7	Muscle weakness	1.12	white matter abnormalities	LP	PS2+PM2	Myasthenic syndrome	Y
n96	M	*TET3*	AD	c.5063G>A (p.R1688H)	het	de novo	2.0	DD, development regression	Na	Na	LP	PS2+PM2	Beck-Fahrner syndrome	Y
n100	F	*CASK*	XLD	chrX:41637634-42135985 deletion	het	de novo	0.5	DD, microcephalus	2.3	microcephaly	P	PVS1+ PS2	Intellectual developmental disorder and microcephaly with pontine and cerebellar hypoplasia	Y

Patient	Gender	Gene	Inheritance pattern	Variant	Zygosity	Origin	Age of onset (years)	Clinical features	Serum lactate (mmol/L)	MRI	ACMG	Evidence	Final clinical diagnosis	Novel variant
n101	F	*GJB2*	AR/AD	c.109G>A (p.V37I)	hom	parental	at birth	DD, hearing loss	3.11	white matter abnormalities	P	PM3+PS4+PP1_S	Autosomal recessive deafness-1A	N
n105	F	*GFAP*	AD	c.235C>T (p.R79C)	het	de novo	3.0	DD, seizure	1.59	white matter abnormalities	P	PS2+PS4+PS3+PM2+PP3+PP4	Alexander disease	N
n106	M	*NF1*	AD	c.8074C>T (p.R2692W)	het	de novo	4.0	DD, ataxia, vertical supranuclear gaze palsy	Na	normal	LP	PS2+PM2+PS2	Neurofibromatosis	Y
n107	F	*CACNA1A*	AD	c.4991G>A (p.R1664Q)	het	de novo	0.7	DD	0.76	cerebellar atrophy	LP	PS2+PM2+PP3	Developmental and epileptic encephalopathy	Y
n109	F	*PRRT2*	AD	c.868dup (p.Y290Lfs*51)	het	maternal	0.4	Seizure	Na	Na	P	PM2_P+PVS1+PS2_M+PS4	Benign familial infantile seizures-2	Y
n110	M	*SCN2A*	AD	c.2150-1G>A	het	de novo	2.0	Seizure, ASD, elevated ethylmalonic acid	Na	Na	P	PM2_P+PVS1+PS2_M	Developmental and epileptic encephalopathy 11	Y
n112	M	*DHDDS*	AR /AD	c.113G>C (p.R38P)	het	de novo	0.8	DD	2.5	normal	LP	PM2_P+PP3+PS2	GDD	N
n117	F	*PURA*	AD	c.32del (p.G11Vfs*67)	het	de novo	0.3	DD, respiratory distress	1.4	Na	LP	PM2_P+PVS1_S+PS2_M	Neurodevelopmental disorder with neonatal respiratory insufficiency, hypotonia, and feeding difficulties	Y
n118	M	*PHOX2B*	AD	chr4:41747992/20A:25A	het	de novo	1.0	Respiratory failure	Na	pallidum involvement	P	PS2_VS+PM1+PM2_P+PP4	Congenital central hypoventilation syndrome	N
n119	F	*ALMS1*	AR	c.12160C>G (p.R4054G); chr2:73827805-73830431 deletion	com.het	paternal	infancy	Multiple organ failure, elevated acylcarnitines	12.5	Na	VUS/VUS	PM2_P/ PVS1_M+PM2_P	Alstrom syndrome	Y
n121	M	*HRAS*	AD	c.35G>C (p.G12A)	het	de novo	infancy	Growth restriction, DD, facial abnormalities, arrhythmia, patent foramen ovale, hypotonia, impaired hearing	Na	basal ganglia involvement	P	PM2_P+PP3+PS4+PS2	Costello syndrome	N
n123	F	*PRRT2*	AD	c.649dup (p.R217Pfs*8)	het	de novo	0.5	Seizure, DD, impaired hearing	1.2	normal	P	PM2_P+PVS1+PS2_M+PS4	Benign familial infantile seizures-2	N
n124	F	*KMT2A*	AD	c.3463T>C (p.C1155R)	het	de novo	at birth	DD, muscle weakness, growth restriction, hypertonia, hypertrichosis	1.1	thin corpus callosum	LP	PM2_P+PP3+PS2_M+PM5	Wiedemann-Steiner syndrome	Y
n125	F	*KAT8*	AD	c.815A>C (p.D272A)	het	de novo	1.0	DD, status epilepsy, hypoglycemia, hypothyroidism	Na	normal	VUS	PM2_P+PP3+PS2_M	Li-Ghorgani-Weisz-Hubshman syndrome	N
n127	F	*MBD5*	AD	c.757A>G (p.S253G)	het	de novo	0.4	DD, seizure, strabismus, hypotonia	Na	white matter abnormalities	VUS	PM2_P+PS2_S	Autosomal dominant intellectual developmental disorder	Y
n128	F	*PCDH19*	XL	c.1160_1178dup (p.R395Afs*12)	het	de novo	3.8	Seizure	1.78	normal	P	PVS1+PM2_P+PS2_S	Developmental and epileptic encephalopathy 9	Y
n131	M	*KCNQ3*	AD	c.1403A>G (p.N468S)	het	maternal	1.4	Seizure, hypoglycemia	1.18	cortical atrophy	VUS	PM2_P+PP3	Benign familial neonatal seizures 2	N
n132	F	*RANBP2*	AD	c.1754C>T (p.T585M)	het	maternal	0.8	Fever, seizure, status epilepsy	6.41	cortical atrophy	VUS	PM2_P+PS2_M	Acute necrotizing encephalopathy	N
n133	M	*CACNA1H*	AD	c.2318G>A (p.G773D)	het	paternal	at birth	Seizure	Na	white matter abnormalities	VUS	PM2_P	Epilepsy	N
n134	M	*SETD5*	AD	c.833G>A (p.R278H)	het	na	0.3	DD, seizure	3.88	normal	VUS	PM2_P+PP3	Intellectual developmental disorder, epilepsy	Y
n135	M	*DMD*	XLR	c.583C>T (p.R195*)	hem	maternal	1.0	Elevated CK	3.74	normal	P	PVS1+PM2_P+PS4	Congenital muscular dystrophy	N
n136	M	*KCNT1*	AD	c.3949C>T (p.R1317*)	het	de novo	2.4	Seizure, development regression	1.16	normal	P	PVS1+PM2_P+PS2_S	Developmental and epileptic encephalopathy	Y

Patient	Gender	Gene	Inheritance pattern	Variant	Zygosity	Origin	Age of onset (years)	Clinical features	Serum lactate (mmol/L)	MRI	ACMG	Evidence	Final clinical diagnosis	Novel variant
													14	
n137	M	*NR0B1*	XLR	c.407dupG (p.Glu137*)	hem	maternal	infancy	Seizure, DD, hypoadrenocorticism	2.21	normal	LP	PVS1+PM2	Congenital adrenal hypoplasia	Y
n138	M	*GJB2*	AR	c.109G>A (p.V37F)	hom	parental	0.3	DD, hearing loss	Na	normal	P	PM3+ PS4+ PP1_S	Autosomal recessive deafness 1A	N
n139	F	*CASK*	AD	c.1711dupT (p.Y571Lfs*9)	het	de novo	0.3	DD	1.05	normal	LP	PVS1+PM2_P	GDD	Y
n157	F	*IQCB1*	AR	c.1090C>T (p.R364*)	hom	parental	8.0	Impaired vision, nystagmus	Na	Na	P	PVS1+PM2_P+PM3_S	Congenital muscular dystrophy	N
n159	M	*CAV3*	AD	c.370T>C (p.C124R); c.277G>A (p.A93T)	com.het	parental	4.0	Elevated CK	Na	Na	VUS/ VUS	PP3/PP3	Congenital myopathy	N/N
n160	M	*HRAS*	AD	c.37G>T (p.G13C)	het	de novo	6.0	Macrocephaly, nystagmus, impaired vision	Na	optic atrophy	P	PP2+PP3+PM1+PM2+PS4_M+PS2_VSt	Costello syndrome	N
n161	M	*STXBP1*	AD	c.1652G>A (p.R551H)	het	de novo	0.8	Seizure, obesity, DD, vision loss, genital malformation	1	white matter abnormalities	P	PM2_P+PM1+PS4+PS2_M+PP2+PP3	Developmental and epileptic encephalopathy 4	N
n163	M	*SCN5A*	AD /AR	c.4892G>A (p.G1631D)	het	de novo	4.1	Arrhythmia	1	Na	LP	PS2+PM2+PP3	Arrhythmia	N
n164	F	*PHOX2B*	AD	chr4:41747990/20A: 25A	het	de novo	0.9	Pulmonary hypertension, central hypoventilation	1	Na	P	PS2+PS4+PM1+PM2	Congenital central hypoventilation syndrome	N
n168	F	*MMACHC*	AR	c.609G>A(p.W203*); c.658_660delAAG (p.K220del)	com.het	parental	infancy	Feeding difficulties, hypotonia,	8.2	basal ganglia involvement	P/P	PM2_P+PM1+PVS1+PM3_VS+PP4/ PM2_P+PM1+PM4+PM3_VS+PP4	Methylmalonic aciduria and homocystinuria, cblC type	N/N
n169	F	*FBP1*	AR	c.170+5G>A; c.755A>T (p.D252V)	com.het	parental	2.0	Vomiting, hypoglycemia, elevated CK, elevated AST	11	normal	VUS/VUS	PM2_P+PP3/ PM2_P+PP3	Fructose-1,6-bisphosphatase deficiency	Y/Y
n170	F	*G6PC*	AR	c.262delG (p.V88Ffs*14); c.326G>A (p.C109Y)	com.het	parental	at birth	Liver disorder	14.05	Na	LP/LP	PM2_P+PVS1/PM2_P+PP3+PM3_S+PP4	Glycogen storage disease Ia	N/N
n171	M	*DMD*	XLR	chrX:31838092-31950344 deletion	hem	maternal	4.3	Elevated CK, hepatomegaly	3.31	Na	P	PVS1+PM2_P+PM1+PP4	Congenital muscular dystrophy	N
n172	M	*SRCAP*	AD	c.7303dupC (p.R2435Pfs*8)	het	de novo	0.3	Growth restriction, feeding difficulties	Na	normal	LP	PVS1_S+PM3+PM2_P	Floating-Harbor syndrome	Y
n173	M	*TPM2*	AD	c.415_417delGAG (p.E139del)	het	de novo	0.5	Muscle weakness, cardiac failure, respiratory failure	Na	Na	P	PM4 + PM6 + PM2_P + PS3 + PS4_P	Nemaline myopathy	Y
n174	F	*CFTR*	AR	c.263T>G (p.L88*); c.2909G>A (p.G970D)	com.het	parental	0.7	Growth restriction, feeding difficulties	3.55	Na	P/P	PVS1+PM3+PM2_P/PM3_S+PM2_P+PS3	Severe malnutrition	N/N
n176	F	*SON*	AD	c.1845_1870del26 (p.G616Sfs*61)	het	de novo	at birth	Growth restriction, DD, hypotonia	4.86	gray matter heterotopia	P	PVS1+PM2_P+PM3	ZTTK syndrome	Y
n177	M	*VPS33B*	AD	c.1030+1G>A; c.1099G>A (p.E367K)	het	de novo	infancy	Growth restriction, DD, hypotonia, cholestasis	3.03	Na	P/VUS	PVS1+PM2_P+PP4 /PM3+PM2_P+PP4 +PP3	Cholestasis	N/Y
n178	F	*G6PC*	AR	c.142C>T (p.P48S); c.310C>T (p.Q104*)	com.het	parental	1.0	DD, hepatomegaly	6.65	normal	VUS/P	PM3+PM2_P+PP3/ PVS1+PM2_P+PM3_P	Glycogen storage disease due to glucose-6-phosphatase deficiency type IA	Y/Y
n181	M	*CUL7*	AR	c.3607+5G>A; c.1252C>T (p.Q418*)	com.het	parental	at birth	Growth restriction, DD	Na	Na	LP/VUS	PVS1+PM2_P/PM3+PM2_P	DD	Y/Y
n183	M	*PRRT2*	AD	c.623_624delinsA (p.S208Yfs*21)	het	paternal	0.40	Seizure	2.36	cortical involvement	LP	PVS1+PM2_P	Benign familial infantile seizures-2	Y
n184	M	*PMM2*	AR	c.337C>T (p.P113S); c.524-8C>G	com.het	paternal	0.3	Seizure	3.7	cerebellar atrophy	VUS/VUS	PM2+PP3/PM2+PP3	Congenital disorder of glycosylation, type Ia	N/Y
n185	F	*FAR1*	AD/AR	c.1499G>A (p.C500Y)	het	de novo	0.8	Motor delay, hypotonia,	1.92	normal	VUS	PM2_P	DD, severe malnutrition,	Y

Patient	Gender	Gene	Inheritance pattern	Variant	Zygosity	Origin	Age of onset (years)	Clinical features	Serum lactate (mmol/L)	MRI	ACMG	Evidence	Final clinical diagnosis	Novel variant
								hyperreflexia					dysphagia, alpha-thalassemia,	
2n1	M	*FLG*	AD	c.5368C>T (p.Q1790*)	het	maternal	0.3	Rash, respiratory distress, liver disorder, elevated acylcarnitine	Na	Na	LP	PVS1_S+PM2_P+PM3	Ichthyosis vulgaris	N
2n2	M	*PYCR2*	AR	c.720_721ins15 (p.A241_N320delinsCTF); c.520dupA (p.S174Kfs*17)	com.het	paternal	0.3	Growth restriction, DD, hypotonia, hyperreflexia, thalassemia	Na	normal	VUS/LP	PM4+PM3+PM2_P/ PVS1+PM2_P	Dyskinesia, GDD, severe malnutrition	Y/Y
		Xp22.33 p21.3		chrX:78263-26560000 duplication		na					P	3C+4C		
2n6	F	*QDPR*	AR	c.661C>T (p.R221*); c.570C>G (p.N190K)	com.het	paternal	10.7	Seizure, cataract, elevated homocysteine	4.77	normal	LP/VUS	PVS1_M+PM3_S+PM2_P/PM2_P+PP3	BH4-deficient hyperphenylalaninemia	N/Y
2n13	M	*ARHGEF6*	XL	c.677C>T (p.P226L)	hem	maternal	at birth	DD, ptosis, hypotonia	Na	normal	VUS	PM2+PP3	GDD	Y
		1q21.3q22		chr1:150725250-156318810 duplication		na					LP	3C		
2n14	M	*TSC2*	AD	c.56T>A (p.L19*)	het	paternal	1.0	Behavioral disorder	Na	normal	P	PVS1+PM2+PP3	Paroxysmal abnormal behavior	Y
		TRIP12	AD	c.2252A>G (p.K751R)	het	de novo					VUS	PM2+PP3		
mn5	F	*MT-RNR1*	maternal	m.1555A>G	99.2%	maternal	0.5	Macrocephaly, hypoglycemia	6.69	cortical abnormalities	P	PS4+PS3+PM9	Glycogen storage disease due to glucose-6-phosphatase deficiency type IA	
		G6PC	AR	c.648G>T (p.L216=)	hom	paternal					P	PS3+PM3_S+PM2_P		N
n114	F	*IDH2*	AD	c.419G>A (p.R140Q)	het	de novo	0.7	DD, cardiac failure, respiratory failure, hypoglycemia	Na	Na	P	PS2_VS+PM2_P+PP3	D-2-hydroxyglutaric aciduria 2	N
n156	F	*CHD2*	AD	c.4602G>C (p.W1534C)	het	de novo	8.0	Paroxysmal loss of consciousness, seizure, feeding difficulties	Na	Na	LP	PS2+PM2_P+PP3	Developmental and epileptic encephalopathy 94	N
n162	M	*MCEE*	AR	c.490A>G (p.K164E); c.40+1G>A	com.het	parental	0.9	Seizure, elevated alanine	1	cyst	VUS/LP	PM2_P+PP3/PVS1+PM2	Coffin-Siris syndrome 1	Y/Y
n90	M	*ARID1B*	AD	c.2703_2704dup (p.A902Efs*13)	he	de novo	at birth	DD, seizure, short stature	1.72	white matter abnormalities	P	PVS1+PS2+PM2_P	Coffin-Siris syndrome	Y
n150	M	*PRIMPOL*	AD	c.965A>C (p.D322A); c.265T>G (p.Y89D)	com.het	parental	7.0	Impaired vision	1.37	optic atrophy	VUS/VUS	PM2_P+PP3/ PM2_P+PP3	Optic atrophy	Y/N

F, female; M, male; AD, autosomal dominant inheritance; XLD, X-linked dominant inheritance; AR, autosomal recessive inheritance; XLR, X-linked recessive inheritance; DD, development delay; ID, intellectual disorder; Na, not available; ACMG, American College of Medical Genetics and Genomics; hom. Homozygote; het, heterozygote; com. het. compound heterozygote; hem, hemizygote; P, pathogenic; LP, likely pathogenic; VUS, variant of uncertain significance; Y, yes; N, no.

Family history: n24, the elder sister was diagnosed with epilepsy at the age of 9 years; n135, the elder brother was diagnosed with hemophagocytic syndrome at the age of 1 year; n161, the father's brother and cousin had a history of epilepsy; n169, the elder sister had fever, seizure and coma, died at the age of 4 years.

Supplemental table 3. Summary of clinical features of patients with non-mitochondria-related copy number variants.

Patients	Gender	Cytoband	Location	Origin	Age of onset (years)	Clinical features	Serum lactate (mmol/L)	MRI	ACMG	Evidence	Final clinical diagnosis
n19	M	17p13.3	chr17:1345462-2400721 deletion	Na	1.00	DD	3.38	white matter abnormalities	P	3C+2A+4B	Miller-Dieker syndrome
n35	F	15q11.2q12	chr15:21200000-29409407 duplication	Na	at birth	DD, seizure, nystagmus	1.62	normal	P	2A+3C	Angelman syndrome
n48	F	1q44	chr1:244535003-246710000 deletion	Na	0.6	DD, seizure, movement disorder	2.6	basal ganglia involvement	LP	2H+4C+5A	GDD, dyskinesia
n72	F	4q12q21	chr14:26830797-41005795 deletion<br>chr14:28767280-39170095 deletion	De novo/<br>De novo	0.3	DD, seizure	2.08	agenesis of corpus callosum	LP/LP	2A+3C+4B+5A/<br>2A+3C+4B+5A	Chromosome 4q deletion syndrome
n99	F	16p13.11	chr16:14957489-16570118 deletion	Na	1.1	Infantile spasms	1.44	cortical atrophy	P	3C+2A+4B	16p13.11 recurrent microdeletion
n103	F	Xp11q21;<br>Xq21q28;<br>Xp22p11	chrX:63185574-63193371 deletion; chrX:85247573-155545276 deletion;<br>chrX:630898-54815682 deletion	De novo/<br>De novo/<br>De novo	0.5	Growth restriction, seizure, hyperglycemia	3.39	normal	P<br>P<br>P	3C+2A+4A+5A<br>3C+2A+4A+5A<br>3C+2A+4A+5A	Turner syndrome
n113	M	22q11.2	chr22:18429208-18659564 duplication	maternal	12.0	Short stature, movement disorder	1.6	normal	P	2A+3B	22q11 duplication syndrome
n120	M	17q11.2	chr17:30971074-31999003 deletion	maternal	0.3	Growth restriction, facial abnormalities	Na	Na	P	3A+2A+5G	NF1-microdeletion syndrome
n158	F	12q13.13	chr12:56923833-62404375 duplication	De novo	8.0	DD	Na	Na	VUS	3A+4C+5G	Chromosome 12q13.13 microduplication

Patients	Gender	Cytoband	Location	Origin	Age of onset (years)	Clinical features	Serum lactate (mmol/L)	MRI	ACMG	Evidence	Final clinical diagnosis
n175	F	22q11.2	chr22:18429208-18659564 deletion	maternal	2.7	Seizure, liver disorder	0.8	normal	P	3C+2A	Inherited metabolic disorder
n182	M	17p11.2	chr17:16744334-20426071 deletion	Na	infancy	DD, feeding difficulties, facial abnormalities	1.26	bilateral lateral ventricles are full in shape	P	3C+2A+4B	Smith Magenis syndrome
n186	M	7p22.1	chr7:6195372-6745369 duplication	Na	2.0	ID	1.38	normal	VUS	3A+4C+5G	ID
2n3	M	8p23.3p23.1	chr8:185003-9652490 deletion	Na	0.3	DD, seizure	3.51	thin corpus callosum	P	3C+2A+4B	8p23.1 deletion syndrome

F, female; M, male; DD, development delay; ID, intellectual disorder; Na, not available; ACMG, American College of Medical Genetics and Genomics; P, pathogenic; LP, likely pathogenic; VUS, variant of uncertain significance.

Family history: n72, the mother has a history of multiple pregnancy loss; n99, the elder sister was diagnosed with febrile seizure at the age of 2 years.
